# Can news-based economic sentiment predict bubbles in precious metal markets?

**DOI:** 10.1186/s40854-022-00341-w

**Published:** 2022-04-06

**Authors:** Aktham Maghyereh, Hussein Abdoh

**Affiliations:** 1 Department of Accounting and Finance, United Arab Emirates University, P.O. Box 15551, Al Ain, UAE; 2 Department of Accounting and Finance, The Citadel: The Military College of South Carolina, Charleston, SC USA

**Keywords:** Asset price bubbles, Market sentiment, Precious metals, G12, G40, C15, C32

## Abstract

This study examines the role of market sentiment in predicting the price bubbles of four strategic metal commodities (gold, silver, palladium, and platinum) from January 1985 to August 2020. It is the first to investigate this topic using sentiment indices, including news-based economic and consumer-based sentiments developed using different methods. We observed the role of sentiment as a reliable indicator of future bubbles for some metal commodities and found that bubbles were regularly concomitant with bearish sentiments for gold and platinum. Moreover, gold and palladium were the only commodities that experienced a bubble during the COVID-19 pandemic. Overall, our findings suggest inclusion of sentiment to the model that predicts the price bubbles of precious metals.

## Introduction

Several studies have highlighted the prevailing role of market sentiment in asset prices (e.g., Baker and Wurgler 2006, 2007; Baker et al. 2012; Brown and Cliff 2004; Kumar and Lee 2006; Yang and Li 2013). These studies suggest that sentiment is an emotional bias that causes deviation from fundamentals. When the deviation far exceeds the fundamental values, price bubbles are created (Stiglitz 1990). Safe-haven asset investments, including precious metals, are likely to increase during crises. As such, sentiment may cause price bubbles of precious metals, making the safe-haven role unwarranted. Baur and Smales (2020) considered the role of four precious metals (gold, silver, palladium, and platinum) in hedging against geopolitical risk (GPR). The authors showed different hedging capabilities of these metals owing to their distinctive supply and demand characteristics. Motivated by their findings, we examine how these precious metals are related to sentiment.

Numerous studies have examined the effects of news-based sentiment on precious metals’ returns and volatility. For instance, Smales (2014, 2015) examined the effect of investor sentiment on the volatility of returns in the gold futures market and found that the sentiment of newswire messages had a significant impact on the returns and volatility of the gold market, whereby negative news had a more significant impact than positive news. Zheng (2015) showed that metal futures returns are asymmetric when responding to investor sentiment shocks. Shen et al. (2017) found that social media sentiment had a significant effect on oil prices. Using social networks from Twitter, Pineiro-Chousa et al. (2018) provided evidence that supports the predictability of investor sentiment and gold returns on S&P 500 returns. Shahzad et al. (2017) and Magayereh and Abdoh (2020) utilized a nonparametric test of causality-in-quantiles and showed that the interdependence between sentiment and six commodities, including gold and silver, differs according to the return quantile.

A growing number of studies have attempted to detect bubbles in precious metal prices. For example, Diba and Grossman (1988) applied frequency analysis and found evidence of bubbles in gold prices. In a similar study, Bertus and Stanhouse (2001) adopted a dynamic factor analysis to document price bubbles in the gold futures markets. Liu and Tang (2010) documented price bubbles in the precious markets. Using the GARCH model, Khalifa et al. (2011) supported the existence of price bubbles in precious metals such as gold and silver. Lucey and O'Connor (2013) adopted the Markov switching ADF tests to confirm the existence of explosive price behaviors in the gold market. Using the supremum augmented Dickey-Fuller (SADF) test, Zhao et al. (2015) recorded five periods of explosive price behaviors in the gold market from 1973 to 2014. Khan and Koseoglu (2020) used the generalized SADF (GSADF) method to test for the explosive behavior of palladium prices. They identified four bubbles in palladium prices during 1994–2020. More recently, Gharib et al. (2021) used the GSADF test to analyze bubbles in the gold prices, and their results detected mildly explosive episodes in July–August 2019.^1^

Although several studies have examined how investor sentiment influences precious metal prices and volatilities, to the best of our knowledge, only one study has examined the sentiment–price bubble relationship in precious metal markets. Pan (2018) investigated the relationship between gold and silver from January 1990 to October 2017. Using indirect market sentiment measures (i.e., the option implied volatility index [VIX]), he found that sentiment, particularly the negative one, increases the probability of bubble occurrence. Although Pan’s study made noteworthy contributions to the literature, it does not address two things. First, it concentrates only on gold and silver and ignores other precious metal markets (i.e., palladium and platinum). Palladium and platinum are unique metals that are more exploited for industrial and manufacturing applications than gold and silver. They represent major input and production costs for manufacturers, making price risk protection a significant strategy in these industries (Khan and Koseoglu 2020). Second, it uses indirect market-based sentiment measures (VIX).^2^ Nevertheless, VIX is not an ideal proxy for investor sentiment because it reflects the investors' mode toward the prospects of stocks and the properties of market volatility.

Hence, given the drawbacks above, our study attempts to extend the literature by exploring the effects of sentiment on four precious metals (gold, silver, palladium, and platinum) from January 1985 to August 2020. This is important because these precious metals are used as core risk-management tools in different industry verticals. Therefore, in this study, we address this gap by investigating how news-based economic sentiment affects the price bubbles of precious metals.

Our study utilizes a model that incorporates the essential drivers behind precious metal prices. Specifically, we control for inflation as it could influence the price of precious metals (particularly gold and silver) used to hedge against the expected decline in the value of money and other financial assets (Labate 1994; Taylor 1998). We include exchange rates in the model because exchange rate variations (i.e., currency risk) may lead investors to pursue the flight-to-safety effect (Hau and Rey 2006; Anderson et al. 2007) toward precious metals. We consider interest rate as an additional control variable that may influence precious metal prices. The expansionary monetary policy reduces interest rates, which may make precious metals more preferred to fixed-income securities. Finally, we capture the impact of economic cycles, which can affect the demand and supply of precious metals. After controlling for these factors, we document the remarkable impact of sentiment on the price bubbles of the precious metals.

We use the news-based economic sentiment index (NESI) developed recently by Shapiro et al. (2020). NESI is constructed based on financial- and economics-related news articles using the lexical approach. The lexical method of sentiment analysis is built on natural language processing, and this process relies on a predefined list of words associated with emotions toward financial and economic news, referred to as emotion lexicons. Compared with market-based (i.e., indirect) and survey-based (i.e., direct) measures of investor sentiment, text-based measures of sentiment extracted from news articles reflect public sentiment in real time (Brochado 2020; Buckman et al. 2020; Maghyereh et al. 2020; Aguilar et al. 2021; among others).^3^ We also use two alternative measures of news sentiment, namely, the Michigan Consumer Sentiment Index (MCSI) and the Investor Sentiment Index (SIBW) developed by Baker and Wurgler (2006). Our results are robust to these alternative sentiment measures.^4^

Econometric literature has suggested several tests to detect asset price bubbles, including variance bounds, West's two-step, and cointegration tests.^5^ However, Gürkaynak (2008) concluded that these tests suffer from model misspecification and do not necessarily capture bubbles. For example, cointegration tests suffer from selected sample bias and can only detect a single bubble episode, thereby failing to detect infrequently collapsing bubbles. Phillips et al. (2011; PWY hereinafter) addressed the drawbacks of the bubble tests mentioned above by proposing a SADF test to detect the existence of single explosive behavior. The PWY method relies on forwarding recursive regressions coupled with sequential right-sided unit root tests.^6^ However, the SADF test does not consistently identify origination and termination dates when price exuberance has more than one episode. Phillips et al. (2015a, 2015b; PSY hereinafter; Phillips and Shi 2019a) proposed a new unit root test, namely, the GSADF, to deal with more than one boom-bust episode that occurs in a single series. PSY generalized the SADF test using large subsamples by changing the start and endpoints of the recursion over a feasible range of flexible windows.^7^ Although the GSADF can effectively detect real-time bubbles in financial markets, it suffers from size distortions caused by heteroscedastic innovations (Harvey et al. 2016, 2019, 2020) and the multiplicity issue of recursive testing (Phillips and Shi 2018).^8^ Phillips and Shi (2020) addressed the potential impact of heteroscedasticity and multiplicity issues of recursive testing algorithms by developing a wild-bootstrap-based implementation of the GSADF test. This study uses Phillips and Shi's (2020) heteroscedasticity and multiplicity-adjusted method (bootstrapped GSADF test) to identify periods of explosive bubbles in precious metals markets.

Our study makes the following three contributions to the literature: First, it tracks the impact of news-based economic sentiment on price bubbles of all primary precious metals (gold, silver, palladium, and platinum) during periods of crises, emphasizing the recent COVID-19 pandemic. Second, we use a news-based economic sentiment measure based on real-time news items gathered by the text processing engine. This measure captures sentiment about current macroeconomic and financial fundamentals and their expectations. Third, the study uses Phillips and Shi's (2020) heteroscedasticity and multiplicity-adjusted method (bootstrapped GSADF test), which has a superior ability to detect jump properties. Fourth, it investigates whether sentiment holds predictive information regarding future price bubbles of precious metals beyond other predictors, such as inflation rate, the Federal Reserve Bank's (the Fed) policy rate, the USD value, the interest rate yield spread, and real economic activity.

Our findings are summarized as follows; first, precious metals experienced multiple episodes of bubbles during the sample period, where most bubbles occurred after the commodity market financialization in the early 2000s. Second, gold is the only asset that has experienced a bubble during the ongoing COVID-19 pandemic (February 2020 to August 2020). Third, gold and silver have the greatest bubble correlations in the precious metal markets. Fourth, all sentiment measures are significant predictors of gold and platinum levels. The price bubbles of these commodities are associated with negative sentiment, suggesting that bearish sentiment induces great investment and demand for the most expensive metals (i.e., gold and platinum), thereby creating pressure on their prices and eventually producing bubbles. As bearish sentiment is likely to occur during market stress, our findings align with the flight-to-safety argument about investing in these metals (Hillier et al. 2006; Baur and McDermott 2010; Maghyereh and Abdoh 2020). Speculation may also contribute to bubble creation when speculators buy gold and platinum at a price above their fundamental values, anticipating a subsequent capital gain. Finally, we evaluate the prediction accuracy of sentiment on commodity price bubbles using the receiver operating characteristic (ROC) curve. We find that the ROC curve can predict gold and silver price bubbles better than those of palladium and platinum. Overall, our findings that the four precious metals exhibit different bubble behaviors could be due to their distinctive supply and demand characteristics. Specifically, palladium and platinum are mainly used in industrial production, whereas gold is primarily used as an investment or store of value with limited industrial demand. Silver possesses dual use as an industrial metal as well as a store of value.

These findings have critical economic and policy implications directed toward understanding the determinants, that is, sentiment, of precious metals' price fluctuations and the differential impact of sentiment on various metal commodities' prices. The findings suggest that long/short strategies that are based on sentiment yield profits from trading gold and platinum when investors purchase these commodities during periods of high sentiment (bullish) and sell them when sentiment is low (bearish). Our findings have important policy implications for countries that export and import these precious metals in large quantities. For instance, global gold producers and consumers can benefit from incorporating sentiment into predicting gold price bubbles. Finally, given the crucial role of precious metals in the economy, policymakers can better forecast the level of economic stability by predicting the price bubbles of these metals.

The remainder of the paper is structured as follows; “Theoretical background” section provides an overview of the theoretical background. “Methodology” section briefly explains the methodology used in this study. “Data and sample” section describes the data used in this study. “Empirical results” section presents the empirical results. Finally, the conclusion is provided in “Conclusions” section.

## Theoretical background

Theoretical studies have presented different definitions of bubbles. Stiglitz (1990) provided a normative definition of a financial bubble:

If the reason that the price is high today is only that investors believe that the selling price will be high tomorrow—when “fundamental” factors do not seem to justify such a price, then a bubble exists. At least in the short run, the high price of the asset is merited because it yields a return (capital gain plus dividend) equal to that of alternative assets. (p. 13)

Under this definition, a financial bubble exists when asset price movements are based on investors' "self-fulfilling prophecies" and not fundamental values.

Lucas’ (1978) asset pricing model is one of the most widely known models used theoretically to analyze and identify multiple bubbles from market fundamentals. Following this model, a substantial body of literature has emerged to improve the theoretical models of financial price bubbles and develop econometric methods for detecting these bubbles (i.e., Shiller et al. 1984; Tirole 1985; Evans 1989; Froot and Obstfeld 1991; Gürkaynak 2008; Doblas-Madrid 2012; Pavlidis et al. 2017; Pavlidis et al. 2018). This article presents a brief conceptual framework based on the present value model of rational commodity pricing. Under the no-arbitrage condition, the commodity price at any time $\left( {P_{t} } \right)$ is given as follows:

1 $$P_{t} = \frac{1}{{\left( {1 + r} \right)}}\left[ {E_{t} \left( {X_{t + 1} } \right) + E_{t} \left( {U_{t + 1} } \right)} \right]$$

where $r$ is the discount factor, which is often referred to as the risk-free interest rate. $E_{t} \left( {} \right)$ denotes the conditional expectation operator, $X_{t + 1}$ and $U_{t + 1}$ indicate the benefits of holding the commodity (expected future capital gains) or expected convenience yields and the unobservable fundamentals component, respectively, in period $t + 1$. The first-order expectation difference in Eq. (1) can be solved using the forwarding iteration as follows:

2 $$P_{t} = P_{t}^{f} + B_{t} = \mathop \sum \limits_{i = 1}^{\infty } \left( {\frac{1}{{\left( {1 + r} \right)}}} \right)^{i} \left[ {E_{t} \left( {X_{t + i} } \right)} \right] + B_{t}$$

where $P_{t}^{f}$ is the fundamental component of commodity price and $B_{t}$ is the bubble component that follows the submartingale property and satisfies the homogeneous expectation equation as follows:

3 $$E_{t} \left( {B_{t + 1} } \right) = \left( {1 + r} \right)B_{t}$$

The first part in Eq. (2) is the “fundamental” commodity price, $P_{t}^{f} = \sum\nolimits_{i = 1}^{\infty } {\left( {\frac{1}{{\left( {1 + r} \right)}}} \right)^{i} \left[ {E_{t} \left( {X_{t + i} } \right)} \right]}$, which is the discounted value of expected future capital gains,^9^ And the second part quantifies the "bubbles," which is the discounted expected future selling price. If investors are willing to pay high prices today because they expect unrealistically to sell the commodity at a higher price on a future date, a bubble commodity price exists (Gürkaynak 2008; Pavlidis et al. 2018). This condition implies that $B_{t} > 0$ in Eq. (2). Thus, psychological behavior drives commodity prices to grow in period $t explosively,$ and this bubble continues to grow until it bursts. In this case, expected future capital gains, ($X_{t}$) and market fundamental, $\left( {P_{t}^{f} } \right)$ are an integrated process of order one, which is $I\left( 1 \right)$. In the absence of bubbles, where $B_{t} = 0$ in Eq. (2), the commodity’s current price is determined by fundamentals, yielding the standard present value model with $P_{t} = P_{t}^{f}$. If $P_{t}^{f}$ is an integrated process of *I*(1), then the current price $\left( {P_{t} } \right){ }$ are also *I*(1) (Homm and Breitung 2012; Areal et al. 2016; Pavlidis et al. 2018; Monschang and Wilfling 2021).

## Methodology

This study focuses on measuring the ability of sentiment to predict explosive bubbles in precious metal markets. Accordingly, we use a two-stage test procedure. In the first stage of analysis, we test the presence and obtain periods of statistically significant explosive pricing behavior by employing Phillips and Shi’s (2020) procedure based on Phillips et al.’s (2015a, 2015b) recursive and rolling window. This stage also includes identifying the beginning and ending dates of those periods. We perform probit models to evaluate whether sentiment can predict bubbles in precious metals' prices in the second stage. This method is briefly summarized here.

### Bubble detection: GSADF test

Following Phillips et al. (2015a, 2015b) and Phillips and Shi (2020), the SADF procedure is based on a standard ADF regression given by

4 $$\Delta P_{t} = \alpha_{{r_{1} ,r_{2} }} + \beta_{{r_{1} ,r_{2} }} P_{t - 1} + \mathop \sum \limits_{i = 1}^{k} \vartheta_{i} \Delta P_{t} + \varepsilon_{t} ,\quad \varepsilon_{t} \sim N\left( {0,\sigma_{{r_{1} ,r_{2} }}^{2} } \right)$$

where $P_{t}$ is the commodity price tested for explosiveness, $r_{1} {\text{and}} r_{2}$ are the start and end points of each subsample period within the window size $r_{w} = r_{2} - r_{1}$. Coefficients $\alpha_{{r_{1} ,r_{2} }}$, $\beta_{{r_{1} ,r_{2} }}$, and $\vartheta_{i}$ are estimated through Ordinary Least Squares (OLS) with a null hypothesis of a unit root $\beta_{{r_{1} ,r_{2} }} = 1$ *vs.* an alternative of a mildly explosive autoregressive coefficient $\beta_{{r_{1} ,r_{2} }}$ $> 1$. $k$ is the lag order included to control for autocorrelation. The SADF test suggested by PWY is based on the forward recursive estimation of the SADF regression in Eq. (4). This estimation has a start point $r_{1}$ fixed at 0, and the endpoint of each sample $r_{2}$ is equal to $r_{w}$, which varies from $r_{0}$ to 1. The SADF statistic is identified as a supremum value of the $ADF_{{r_{2} }}$ sequence for $r_{2} { } \in \left[ {r_{0} ,1} \right]$ and is represented by $SADF_{{r_{0} }}$. Thus, the SADF statistic is as follows:

5 $$SADF_{{r_{0} }} = \underbrace {\sup }_{{r_{2} \in \left[ {r_{0} ,1} \right]}}\left\{ {ADF_{{r_{2} }} } \right\}$$

PSY proposed the GSADF to improve the ability to detect multiple episodes of bubbles by allowing start point $r_{1}$ in the SADF regression model (4) to vary within the range $\left[ {0, r_{2} - r_{0} } \right]$, thereby doubling the recursive subsample structure. Following PSY’s recommendation, the minimum window size required to initiate regression $r_{0}$ is set to $0.01 + 1.8/\sqrt T$, and a fixed lag order of $k = 0$ is set for Eq. (4). The GSADF statistic denoted by $GSADF_{{r_{0} }}$ is as follows:

6 $$GSADF_{{r_{0} }} = \underbrace {\sup }_{{_{{r_{1} \in \left[ {0,r_{2} - r_{0} } \right]}}^{{r_{2} \in \left[ {r_{0} ,1} \right]}} }}\left\{ {ADF_{{r_{1} }}^{{r_{2} }} } \right\}$$

The existence of bubbles within the series can be tested by comparing the $GSADF_{{r_{0} }}$ statistic with the corresponding right-tail critical values obtained from Phillips and Shi’s (2020) wild-bootstrap procedure described below. If the $GSADF_{{r_{0} }}$ statistic is greater than the right-tail critical value, then we can confirm that the sample period has at least one bubble.^10^

Next, we use a backward SADF (BSADF) to identify the windows in which the bubbles exist in the data. The BSADF test statistic sequence provides the origination and termination dates of the identified bubble episodes. The BSADF statistics denoted by $BSADF_{{r_{2} }}$ are defined as follows:

7 $$BSADF_{{r_{2} }} = \underbrace {\sup }_{{r_{1} \in \left[ {0,r_{2} - r_{0} } \right],r_{2} = \left[ {r_{0} ,1} \right]}}\left\{ {ADF_{{r_{1} }}^{{r_{2} }} } \right\}$$

Based on the sequence of the BSADF test statistic, the estimated origination and termination dates of a bubble denoted by $\hat{r}_{e}$ and $\hat{r}_{f}$, respectively, are as follows:

8 $$\hat{r}_{e} = \underbrace {\inf }_{{r_{2} = \left[ {r_{0} ,1} \right]}}\left\{ {r_{2} :BSADF_{{r_{2} }} > cv_{{r_{2} }} \left( {\beta_{T} } \right)} \right\}$$

9 $$\hat{r}_{f} = \underbrace {\inf }_{{r_{2} = \left[ {\hat{r}_{e} ,1} \right]}}\left\{ {r_{2} :BSADF_{{r_{2} }} < cv_{{r_{2} }} \left( {\beta_{T} } \right)} \right\}$$

where $cv_{{r_{2} }} \left( {\beta_{T} } \right)$ is the $100\left( {1 - \beta_{T} } \right)\%$ right-tail critical value of the $BSADF_{{r_{2} }}$ statistic based on the sample size $T_{{r_{2} }}$.

Recently, Phillips and Shi (2020) suggested using a wild-bootstrap re-sampling scheme to alleviate the potential effect of unconditional heteroscedasticity and multiplicity issues in the PSY procedure because these could cause frequent spurious identification of a bubble. Following these steps, we draw statistical inferences of the PSY statistics. The wild-bootstrap resampling scheme comprises the following five steps:

*Step 1*.: Run the regression in Eq. (4) on the entire sample period under the imposition of the null hypothesis $\beta = 0$ and obtain the estimated residual $\hat{\epsilon }_{t}$.
*Step 2*.: For the sample size $\tau_{0} + \tau_{b} + 1$ (where $\tau_{0} = \left[ {Tr_{0} } \right]$ and $\tau_{b}$ is the number of observations in the window), construct the bootstrap sample by $\Delta P_{t}^{b} = \mathop \sum \limits_{j = 1}^{p} \vartheta_{j} \Delta P_{t - j}^{b} + \epsilon_{t}^{p}$ with initial values ${ }P_{i}^{b} = P_{i}$, $i = 1, \ldots ,j + 1$. Coefficients $\vartheta_{j}$ are obtained from the fitted regression in Step 1 using the OLS estimates.
*Step 3*.: Calculate the bootstrap BSADF test statistic denoted by $\left\{ {{\text{BSADF}}_{b}^{*} } \right\}_{{t = \tau_{0} }}^{{\tau_{0} + \tau_{b} + 1}}$ using the bootstrap sample series with a maximum value statistics as follows: ${\mathcal{M}}_{t}^{b} = \underbrace {\max }_{{t \in \left[ {\tau_{0} ,\tau_{0} + \tau_{b} + 1} \right]}}({\text{BSADF}}_{b}^{*} )$.
*Step 4*.: Repeat Steps 1–3 for *B* = 999 times to generate a sample of bootstrapped ${\text{BSADF}}_{b}^{*}$.
*Step 5*.: The critical bootstrap value of the PSY procedure denoted by $cv_{\alpha }^{b}$ is given by $\alpha = 95\%$ percentiles, which is calculated from $\left\{ {{\mathcal{M}}_{t}^{b} } \right\}_{b = 1}^{B}$ replications.^11^

### Bubble predictor: probit approach

We examine the extent to which sentiment affects the likelihood of bubbles in precious metal markets from January 1985 to August 2020 using a multivariate probit model as follows:

10 $$p_{i} = \Pr \left( {R_{t} = 1} \right) = f\left( {\beta_{0} + \beta_{1} S_{t - 1} + \beta_{i} X_{it - t} + \varepsilon_{t} } \right)$$

11 $$R_{t} = \left\{ {\begin{array}{*{20}c} {1,} & {if\,\left\{ {{\text{BSADF}}_{b}^{*} } \right\}_{{t = \tau_{0} }}^{{\tau_{0} + \tau_{b} + 1}} \ge cv_{\alpha }^{b} } \\ {0,} & { if\,\left\{ {{\text{BSADF}}_{b}^{*} } \right\}_{{t = \tau_{0} }}^{{\tau_{0} + \tau_{b} + 1}} < cv_{\alpha }^{b} } \\ \end{array} } \right.$$

where $Pr\left( . \right)$ is the probability of a bubble occurrence, and $R_{t}$ is a binary dependent variable that equals one if a bubble is detected (the BSADF sequence exceeds the corresponding bootstrap critical value), and 0 otherwise. $S_{t - 1}$ is the sentiment index at time $t - 1$. $X_{it}$ is a set of well-established control variables that affect the bubble behaviors of precious metal prices, including inflation, the USD exchange rate, and the Fed’s policy rate. $\varepsilon_{t}$ is the error term $\varepsilon_{t} \sim iid\left( {0,\sigma^{2} } \right).$ We observed all the above variables at time *t* − 1 to investigate their predictive power.

Economic theory predicts that inflation directly influences precious metal prices in regards to the control factors. When investors expect high future inflation rates, they become more attracted to precious metals, (particularly gold and silver) which hedge against the expected decline in the value of money and other financial assets (Labate 1994; Taylor 1998). This situation leads to a rally to purchase precious metals, consequently creating potential market price bubbles. Several empirical studies have confirmed the strong relationship between inflation and precious metal prices, such as Harmston (1998), Tully and Lucey (2007), Zhu et al. (2018), and Salisu et al. (2019). Other studies indicate the role of precious metals as a hedge against inflation, such as Adrangi et al. (2003), Lucey and Li (2015), Bampinas and Panagiotidis (2015), Zhu et al. (2018), and Salisu et al. (2019).

In addition to inflation, precious metal prices are also affected by exchange rates. Theoretically, exchange rate variations (i.e., currency risk) may lead investors to pursue the flight-to-safety effect.^12^ In turn, this condition induces investors to pursue safe-haven assets (Hau and Rey 2006; Anderson et al. 2007), particularly precious metals. Numerous studies have shown that the USD exchange rate and precious metal prices are negatively correlated (e.g., Sjaastad and Scacciavillani 1996; Capie et al. 2005; Pukthuanthong and Roll 2011; Harris and Shen 2017; Huang et al. 2019). Other studies provide evidence of the safe-haven properties of precious metals (Pierdzioch et al. 2016; Bedoui et al. 2020; Mensi et al. 2020). This study uses the trade-weighted USD index (USDI; major currencies) as a proxy for the exchange rate (e.g., Hammoudeh et al. 2015; Huang et al. 2019).

We consider interest rate as an additional control variable that may influence precious metal prices. Theoretically, expansionary monetary policy reduces interest rates, which may make precious metals more desirable for investors than fixed-income securities. Thus, low interest rates increase commodity prices (Frankel 2006). Agnello et al. (2020), a recent empirical study, showed that an increase in interest rates would shorten commodity price booms and lengthen busts.^13^ Following Thorbecke and Zhang (2009), Hammoudeh et al. (2015), and Huang et al. (2019), we use the Fed’s policy rate as a proxy for interest rate. Following Batten et al. (2010), Karali and Power (2013), Frankel (2014); Gruber and Vigfusson (2013), Magrini and Donmez (2013), Mo et al. (2018), among others, we also used the interest rate yield spread between the 10-year and 2-year US bonds "Treasury Constant Maturity Rate" to proxy for the slope of the yield curve. The rationale for using this variable is that term spread is closely related to the business cycle (Collin-Dufresne et al. 2001; Ang et al. 2006). A negative (positive) spread signals a near-future economic recession (expansion). Therefore, we expect to find increasing precious metal prices as the spread narrows.

The demand and supply of precious metals are also affected by real economic activities. Arango et al. (2012), Varadi (2013), Baur and Tran (2014), Kucher and McCoskey (2016), Fernandez (2016), and Duarte et al. (2021), among others, argue that commodity prices are highly linked to business cycles. To capture the impact of the economic cycles, we consider the monthly index of global real economic activity in industrial commodity markets, which was originally developed by Kilian (2009) and recently corrected by Kilian (2019). This business cycle index is based on the dry cargo single voyage ocean freight rates, and it captures shifts in the demand for industrial commodities.

## Data and sample

The monthly data set used in this study includes an index of sentiment and four precious metals (gold, silver, palladium, and platinum) from January 1985 to August 2020 (a total of 420 observations). All precious metal data were obtained from the Thomson Reuters DataStream database. Inflation, USD exchange rate, and the Fed’s policy rate are obtained from Federal Reserve Economic Data of the St. Louis Fed (https://fred.stlouisfed.org/).

We empirically consider the impact of sentiment on various precious metals using the news-based economic sentiment index (NESI) of Shapiro et al. (2020).^14^ NESI is constructed based on economics-related news articles using the lexical approach. The lexical method of sentiment analysis is built on natural language processing. This process relies on a predefined list of words associated with emotions toward financial and economic news, referred to as emotion lexicons. This approach measures the emotional content of a large corpus of economics/finance news articles based on the spread of negative vs. positive words in the corpus, where positive words are assigned a score of 1 and negative words a score of − 1. The data include more than 238,685 economic and financial news articles from 16 major newspapers. The overall time series economic news sentiment score is normalized, therefore, it continuously ranges from − 1 to 0 and from 0 to 1, indicating negative and positive economic sentiments that measure the real-time agents' degree of pessimism and optimism over the state of the economy, respectively.^15^ In this regard, when investors are optimistic about the state of the economy, they may overreact to pleasant financial and economic news (i.e., behave in an overconfident manner), potentially leading to jumps in precious metals prices. Conversely, in the case of pessimistic sentiment, demand decreases, causing precious metals prices to decrease.

We use two alternate news economic sentiment measures, namely, the Michigan Consumer Sentiment Index (MCSI) and the investor sentiment index (SIBW) developed by Baker and Wurgler (2006). The MCSI is constructed based on a monthly survey containing five core questions representing current and future economic and financial expectations. MCSI is measured by subtracting the proportion of consumers who provide favorable replies from the proportion of unfavorable ones.^16^ Therefore, the index measures the degree of public confidence (optimism) over the state of the economy. A high index value indicates high confidence in future economic and financial states. MCSI data were obtained from St. Louis Fed (https://fred.stlouisfed.org/series/UMCSENT). SIBW is a market-based measure constructed by combining several single market-based proxies into a composite sentiment index using the principal component analysis (Baker and Wurgler 2006).^17^ Although the Baker and Wurgler sentiment index is widely used in literature, its major drawback from the standpoint of this study is that it is predominantly oriented toward the equity market. However, the equity market continues to be the most liquid market. Hence, proxies from this market can be representative of general economic and financial sentiment (Gao and Süssb 2015). Nevertheless, in this study, we supplement this measure with our main sentiment indicator (i.e., the NESI) to better reflect the general mood of the current and future general economic and financial states. Table 1 presents the descriptions of the variables.

**Table 1:** Variable descriptions

Variable	Description	Source
NESI	The news-based economic sentiment index	Developed by Shapiro, Hale, Sudhof, and Wilson (2020), available at San Francisco Fed’s website: https://www.frbsf.org/economicresearch/indicators-data/daily-news-sentiment-index/
MCSI	Consumer Sentiment Index	Developed by University of Michigan, retrieved from FRED, Federal Reserve Bank of St. Louis: https://fred.stlouisfed.org/series/UMCSENT
SIBW	Sentiment index of Baker and Wurgler (2006)	Developed by Baker and Wurgler (2006), available at: http://people.stern.nyu.edu/jwurgler/
Inflation	Measured as the monthly percentage change in Consumer Price Index (CPI)	Federal Reserve Economic Data of the Federal Reserve Bank of St. Louis
USDI	USD dollar index	Federal Reserve Economic Data of the Federal Reserve Bank of St. Louis
EFR	Effective Federal Funds Rate	Federal Reserve Economic Data of the Federal Reserve Bank of St. Louis
T-Spread	Interest rate yield spread measured as the difference between 10-year and 2-year US bonds constant maturity rate	Federal Reserve Economic Data of the Federal Reserve Bank of St. Louis
GEA	Kilian global real economic activity index	Federal Reserve Bank of Dallas

Table 2 shows the statistical characteristics of the sentiment measures and macroeconomic indicators, including inflation, USD value, Fed's policy rate, interest rate yield spread, and Kilian's (2009) global real economic activity index. Most sentiment measures are negatively skewed, whereas macroeconomic indicators are positively skewed. The MCSI standard deviation is greater than the remaining sentiment indices ( NESI and SIBW), and the USDI standard deviation, at 12.41, is the greatest. The kurtosis of the sentiment measures ( NESI and SIBW), inflation, and USDI are greater than three, indicating a leptokurtic distribution. Additionally, these variables are not normally distributed, as shown by the Jarque–Bera test. This finding rejects the null hypothesis for the Gaussian distribution at the 1% significance level.

**Table 2:** Descriptive statistics of variables, January 1985–August 2020

	NESI	MCSI	SIBW	Inflation	USDI	EFR	T-Spread	GEA
Mean	0.0772	88.0904	0.1383	0.0012	89.7878	3.6156	1.0955	− 0.0970
Median	0.0866	90.9000	− 0.0289	− 0.0022	89.0798	3.7300	1.0350	− 7.5000
Maximum	0.6015	112.0000	2.9387	0.2457	143.9059	9.8500	2.8300	190.810
Minimum	− 0.6253	55.3000	− 0.9417	− 0.1807	69.0608	0.0700	− 0.4100	− 159.47
Std. Dev	0.2255	11.7058	0.6213	0.0470	12.4077	2.8077	0.8478	58.6832
Skewness	− 0.3912	− 0.5822	1.4577	0.2359	1.2007	0.2091	0.2376	0.8926
Kurtosis	3.1131	2.9896	6.2592	5.7211	5.9610	1.8156	1.9000	4.1265
Jarque–Bera test	10.5***	22.99***	324.2***	129.3***	246.4***	26.7***	25.6***	79.4***
	(0.0050)	(0.0000)	(0.0000)	(0.0000)	(0.0000)	(0.0000)	(0.0000)	(0.0000)

*p* values are given in brackets. *** indicates significance at 1% level

The stationarity of variables must be tested before estimating the probit model to avoid spurious results because of non-stationarity (Regenwetter and Davis-Stober 2018). Table 3 shows the stationarity of our variables using the augmented Dickey-Fuller (ADF) test and Phillips–Perron (PP) test. Both tests reject the null hypothesis at the conventional level of statistical significance, indicating that the variables are stationary. The ADF and PP unit root tests have low power if structural breaks occur in the time series (Pierre 1989; Nazlioglu 2011; Awartani et al. 2020). To improve the power of the unit root test, we further use the procedure developed by Zivot and Andrews (1992) to test the null of the unit root against the stationary break alternative. The results of the Zivot and Andrews unit root tests are presented in Table 4. The t-statistics show that all our variables are stationary with a breakpoint, which is confirmed for all variables using the ADF test and the PP test. The table also shows that the estimated breakpoints for all variables occurred around the global financial crisis (GFC) of 2007–2008.

**Table 3:** ADF and PP stationary tests

	ADF test		PP test	
	Including intercept	Including intercept and trend	Including intercept	Including intercept and trend
NESI	− 5.6730***	− 5.7730***	− 5.5821***	− 5.7052***
	(0.0050)	(0.0000)	(0.0000)	(0.0000)
MCSI	− 3.4673***	− 3.5441**	− 3.1910**	− 3.2927*
	(0.0093)	(0.0361)	(0.0212)	(0.0688)
SIBW	− 3.4034**	− 4.7582***	− 3.2307**	− 3.4661**
	(0.0141)	(0.0006)	(0.0190)	(0.0445)
Inflation	− 16.6796***	− 16.6604***	− 22.9012***	− 22.8348***
	(0.0000)	(0.0000)	(0.0000)	(0.0000)
USDI	− 4.8289***	− 4.2260***	− 4.0976***	− 3.3947*
	(0.0001)	(0.0044)	(0.0011)	(0.0534)
EFR	− 2.8337**	− 3.5288**	− 2.9820*	− 3.7906**
	(0.0463)	(0.0376)	(0.0893)	(0.0334)
T-Spread	− 2.8572**	− 3.5756**	− 2.9329*	− 3.0242*
	(0.0465)	(0.0291)	(0.0933)	(0.0801)
GEA	− 4.0436***	− 4.0410***	− 3.4120**	− 3.4094*
	(0.0013)	(0.0082)	(0.0111)	(0.0514)

Table reports the Augmented Dickey-Fuller (ADF) and Phillips–Perron (PP) tests for stationarity. *p* values are given in brackets. *, **, and *** indicate significance at 10%, 5%, and 1% levels, respectively

**Table 4:** Zivot-Andrews (ZA) stationary test

	Including intercept		Including intercept and trend	
	Test statistics	Break date	Test statistics	Break date
NESI	− 4.6953**	2007:M06	− 4.7512**	2007:M08
	(0.0342)		(0.0237)	
MCSI	− 4.3398***	2007:M08	− 4.8534***	2007:M08
	(0.0052)		(0.0025)	
SIBW	− 4.4101***	2006:M10	− 4.6011***	2006:M10
	(0.0008)		(0.0000)	
Inflation	− 4.8220***	2008:M10	− 4.8519***	2008:M09
	(0.0089)		(0.0008)	
USDI	− 4.9993***	2008:M02	− 4.9500***	2007:M08
	(0.0001)		(0.0001)	
EFR	− 3.4722**	2008:M02	− 3.7061***	2008:M10
	(0.0166)		(0.0040)	
T-Spread	− 3.1510***	2007:M08	− 3.5337***	2007:M08
	(0.0087)		(0.0037)	
GEA	− 4.8605***	2007:M06	− 4.8512***	2007:M07
	(0.0004)		(0.0018)	

The table reports the Zivot-Andrews (ZA) statistics, which allows for both a structural break in intercept, trend or both. The null hypothesis of the ZA test is that the series has a unit root with a structural break(s) against the alternative hypothesis that they are stationary with a break(s). *p* values are given in brackets. *, **, and *** indicate significance at 10%, 5%, and 1% levels, respectively

Table 5 shows the correlation values between explanatory variables. NESI is highly positively correlated with MCSI (0.642) and SIBW (0.527). The inflation rate is not significantly correlated with any of these sentiment indices. Nevertheless, the Fed's policy rate is positively related to sentiment, supporting the effect of monetary policy decisions on sentiment (e.g., Kurov 2010). As expected, inflation and the Fed's policy rate have a positive relationship because the Fed aims to reduce inflation by contracting money supply in the economy through a high policy rate. An increased rate may also attract investments in interest-bearing assets (e.g., bonds), thereby raising the demand and value of USD.

**Table 5:** Correlations between explanatory variables

	NESI	MCSI	SIBW	Inflation	USDI	EFR	T-Spread	GEA
NESI	1.0000							
MCSI	0.6424***	1.0000						
	(0.0000)							
SIBW	0.5276***	0.3062***	1.0000					
	(0.0009)	(0.0000)						
inflation	0.0497	0.0511	0.0385	1.0000				
	(0.9958)	(0.9945)	(0.9998)					
USDI	0.2407***	0.5425***	0.5176***	0.0179	1.0000			
	(0.0000)	(0.0000)	(0.0000)	(0.7008)				
EFR	0.2252***	0.3961***	0.3991***	0.2757***	0.5104***			
	(0.0000)	(0.0000)	(0.0000)	(0.0000)	(0.0000)	1.0000		
T-Spread	− 0.3415***	− 0.6049***	− 0.2738***	− 0.1188***	− 0.3435***	− 0.6767***	1.0000	
	(0.0000)	(0.0000)	(0.0000)	(0.0165)	(0.0000)	(0.0000)		
GEA	0.2147*	0.2188***	0.1613***	0.1681***	− 0.4092***	0.1094**	− 0.2516**	1.0000
	(0.0676)	(0.0000)	(0.0011)	(0.0007)	(0.0000)	(0.0274)	(0.0298)	

*p* values are given in brackets. *p* values are given in brackets. *, **, and *** indicate significance at 10%, 5%, and 1% levels, respectively

## Empirical results

### Bubble detection

**Fig. 1:**
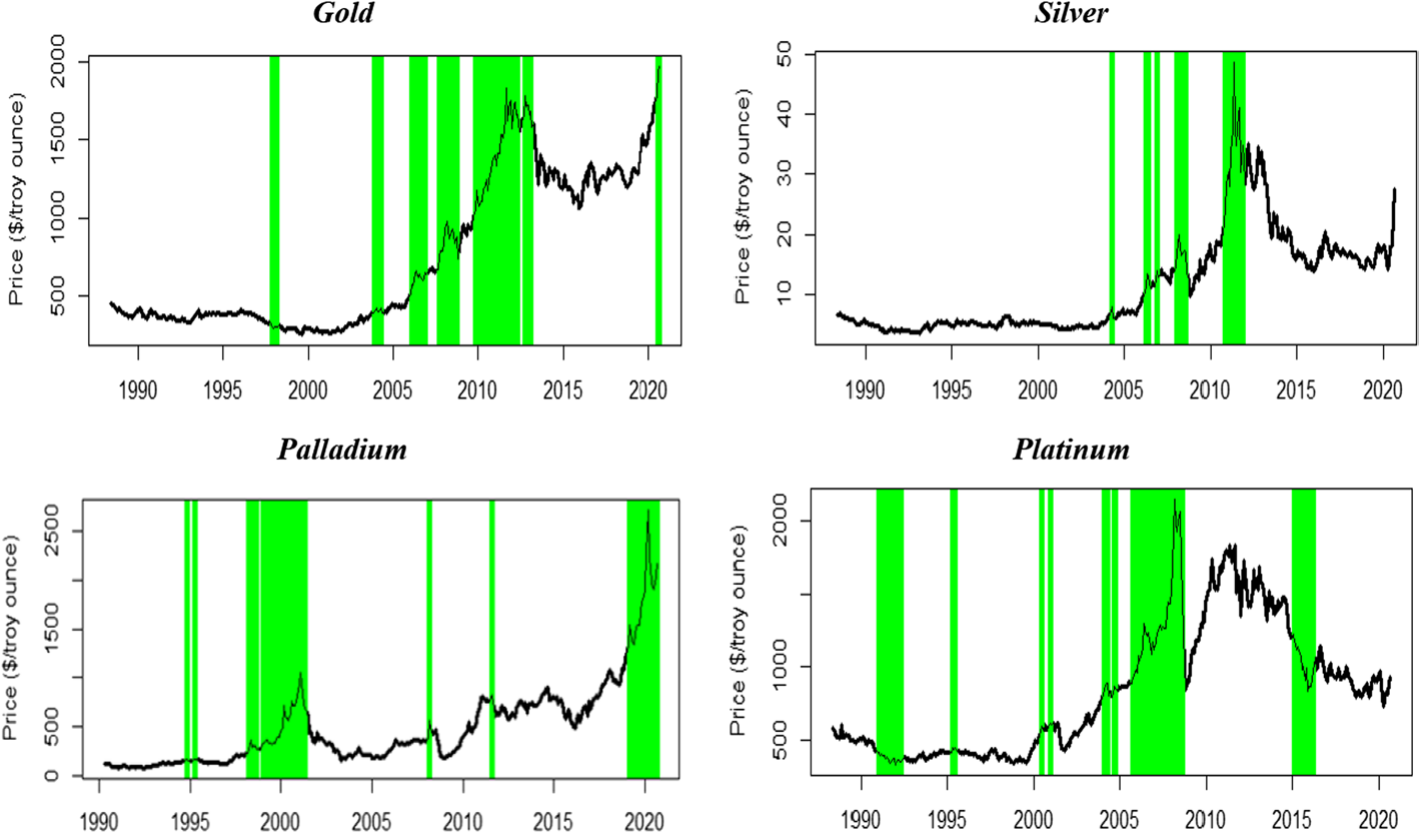
Bubbles and crisis periods in precious metal prices, GSADF test. Notes: The solid lines are the price of the precious metal commodity, and the green-shaded areas indicate bubble periods. The shaded areas are identified when the BSADF statistic exceeds the corresponding 95% bootstrapped critical value. The 95% bootstrapped critical values are obtained from 999 bootstrap replications

Figure 1 plots metal commodity prices (solid black line) and bubble periods, where the PSY statistic exceeds its 95% bootstrapped critical value (green-shaded areas). We observe a sharp rise in precious metal prices (gold, silver, and platinum) during the global financial crises (2008–2009). Additionally, gold and silver prices increased sharply during the European sovereign debt crisis (2010–2012). Conversely, palladium prices did not experience a sharp increase during these crises. Palladium is more exploited for industrial and manufacturing applications than other ferrous metals such as gold and silver. For example, the palladium bubble in 1998:M0-2001: M04 was due to massive supply disruptions in major palladium producers (i.e., Russia) and a surge in demand due to the technology boom.

We date-stamped the start and the end of each price bubble period for each precious metal in Table 6. We used the 95% bootstrapped critical values obtained from 999 bootstraps from Monte Carlo simulations. Most episodes are short-lived (i.e., persisting under 12 months) and do not correspond to the same periods for the different metals considered. Gold witnessed a price bubble during the GFC (2007–2009), contrary to silver, palladium, and platinum.

**Table 6:** Bubble origination and collapse dates in precious metal prices, BSADF test

	Gold		Silver		Palladium		Platinum	
	Start	End	Start	End	Start	End	Start	End
1	1997: M11	1998: M02	2004: M03	2004: M04	1994: M10	1995: M03	1990: M12	1992: M04
2	2003: M11	2003: M12	2006: M03	2006: M05	1998: M03	2001: M04	1995: M04	1995: M05
3	2004: M03	2004: M04	2006: M11	2006: M12	2008: M02	2008: M04	1995: M06	1995: M07
4	2006: M01	2006: M07	2008: M01	2008: M07	2011: M07	2011: M08	2000: M06	2000: M07
5	2006: M11	2006: M12	2010: M10	2011: M08	2019: M02	2020: M08	2000: M12	2001: M01
6	2007: M09	2008: M07	2011: M10	2011: M11			2004: M01	2004: M03
7	2008: M09	2008: M10					2004: M08	2004: M09
8	2009: M10	2012: M04					2005: M09	2008: M08
9	2012: M09	2013: M01					2015: M02	2016: M03
10	2020: M02	2020: M08						
*BSADF critical values*								
90%	1.719248		0.8885958		-0.07549508		0.17888688	
95%	2.390631		1.2735069		0.54208558		0.63439393	
99%	3.050253		1.6195983		1.87499433		1.67793629	

This table reports bubble origination and termination dates identified with 95% critical values obtained by the Wald bootstrap procedure of Phillips and Shi (2020). The 95% bootstrapped critical values are obtained from 999 bootstrap replications

Gold is the only precious metal that experienced a bubble during the COVID-19 pandemic (February 2020 to August 2020). This finding can be attributed to the greater safe-haven role of gold compared to other precious metals, supported by unprecedented monetary stimulus and interest rate cuts by major central banks around the globe to cushion the economic impact of the COVID-19 pandemic. Gold is used more as a store of value or investment than silver and other precious metals. In construction, precious metals such as silver, platinum, and palladium are much more used in manufacturing, and are therefore more driven by industrial demand.

Silver and platinum did not experience any bubbles after 2011, 2012, and 2016, respectively. The findings showed multiple episodes of bubbles in each of these metals. Gold witnessed the greatest number of bubble episodes, whereas palladium witnessed the most bubbles during the period 2019:M02-2020. In 2019, palladium outperformed other precious metals, and prices increased by approximately 60%, mainly due to the low supply. Furthermore, supply disruption during the COVID-19 pandemic led to palladium prices jumping to $2719 per ounce by the end of March 2020. Finally, most of these bubbles occurred after the commodity market financialization in the early 2000s. Gold and platinum experienced the longest bubble periods from October 2009 to April 2012 and from September 2005 to August 2008, respectively.

### Bubble and news-based economic sentiment

This section presents the role of sentiment in predicting price bubbles for precious metals. We show the correlation between bubbles across precious metal markets, as shown in Table 7, before approaching the main regression in this section. Bubbles in the gold and silver markets have a greater connection than other markets. The correlation between gold and silver bubbles is approximately 0.5, whereas that between gold or silver and palladium and platinum does not exceed 0.3. The relatively high frequency of gold bubbles suggests that gold market participants may risk large losses when these bubbles burst.^18^

**Table 7:** Correlations between bubbles across precious metal markets

	Gold	Silver	Palladium	Platinum
Gold	1.0000			
Silver	0.4932***	1.0000		
	(0.0000)			
Palladium	0.1091**	0.2056***	1.0000	
	(0.0241)	(0.0000)		
Platinum	0.2091**	0.2056***	0.0943*	1.0000
	(0.0241)	(0.0000)	(0.07744)	

*p* values are given in brackets. *, **, and *** indicate significance at 10%, 5%, and 1% levels, respectively

Table 8 lists the measurements of the impact of sentiment on the probability of bubble occurrence. We estimate a probit model with a dependent binary variable *Bubble* that equals 1 when the PSY (the supremum of the estimated ADF) statistic for the relevant observation is above the generated critical value for the regarded metal (i.e., a bubble exists) and 0 otherwise (i.e., a bubble does not exist). The last row of Table 8 shows that the model identifies well whether the metal is experiencing a bubble. This observation is also supported by the non-significance of the Hosmer–Lemeshow test, thereby indicating a good-fit regression model. Panel A shows that the lagged NESI is a significant predictor of only gold and platinum. The bearish sentiment (or pessimism) precedes these metals bubbles as indicated by the negative coefficient of NESI. These findings suggest that during the bad economic condition (i.e., crises), low sentiment shifts a greater demand toward safe-haven commodities (i.e., gold and platinum), raising their prices and creating bubbles. Table 6 illustrates that these bubbles are concentrated during economic crises.

**Table 8:** Estimation results using news-based economic sentiment index

	Dependent variable: Bubble			
	Gold	Silver	Palladium	Platinum
*Panel A: Probit models*				
$NESI_{t - 1}$	− 1.5088***	0.2503	0.7318	− 1.0746***
	(0.0020)	(0.7400)	(0.2810)	(0.0000)
$Inflation_{t - 1}$	0.3800**	0.1281*	0.5802***	0.2080***
	(0.0200)	(0.0940)	(0.0000)	(0.0000)
$US{\text{DI}}_{t - 1}$	− 1.2551***	− 0.2018**	− 0.0546***	− 0.6100***
	(0.0030)	(0.0120)	(0.0080)	(0.0021)
${\text{EFR}}_{t - 1}$	− 0.1560**	0.2178	− 0.4890***	− 0.2283***
	(0.0240)	(0.2110)	(0.0000)	(0.0000)
$T - Spread_{t - 1}$	− 0.4356*	− 1.2005**	− 0.3744**	− 0.4551***
	(0.0780)	(0.0120)	(0.0311)	(0.0200)
${\text{GEA}}_{t - 1}$	0.0102***	0.0060**	0.0045**	0.0062***
	(0.0000)	(0.0200)	(0.0138)	(0.0000)
$Constant$	0.3189***	0.3698***	0.5793***	0.2435***
	(0.0000)	(0.0029)	(0.0000)	(0.0000)
*Panel B: Conditional marginal effects*				
$NESI_{t - 1}$	− 0.2675***	0.0170	0.0973	− 0.2425***
	(0.0060)	(0.7430)	(0.2440)	(0.0010)
$Inflation_{t - 1}$	0.2447**	0.0871*	0.7419***	0.0498***
	(0.0360)	(0.0945)	(0.0000)	(0.0000)
$US{\text{DI}}_{t - 1}$	− 0.2225***	− 0.0137***	− 0.0073***	− 0.0226***
	(0.0010)	(0.0020)	(0.0010)	(0.0020)
${\text{EFR}}_{t - 1}$	− 0.0277**	0.0148	− 0.0650***	− 0.0515***
	(0.0120)	(0.2070)	(0.0000)	(0.0000)
$T - Spread_{t - 1}$	− 0.0772**	− 0.0816**	− 0.0498**	− 0.1027**
	(0.0750)	(0.0160)	(0.0292)	(0.0220)
${\text{GEA}}_{t - 1}$	0.0018***	0.0004**	0.0006**	0.0014***
	(0.0000)	(0.0330)	(0.0135)	(0.0000)
$Observations$	420	420	420	420
McFadden's pseud-R^2^	0.8334	0.5714	0.4757	0.6726
Log-likelihood	− 117.6715	− 48.2477	− 92.6440	− 162.2984
Hosmer–Lemeshow test	7.09	8.12	6.67	11.97
	(0.3690)	(0.1887)	(0.1598)	(0.2176)
${\text{Correct bubble}}$	84.13%	75.00%	74.42%	96.30%
${\text{Correct no}} - {\text{bubble}}$	96.99%	96.21%	91.67%	86.88%
${\text{Correct }}\left( {{\text{classified}}} \right){\text{ overall}}$	92.36%	95.59%	91.91%	87.50%

The dependent variable is a binary that equals 1 (bubble dates) and 0 (none-bubble dates) identified by the GSADF procedure. Panels A and B report the results of the probit regressions and conditional marginal effects of a unit change in the mean value of the explanatory variables on the probability of a bubble. The Hosmer–Lemeshow test is a statistical test for goodness of fit for probit regressions, which follows an $\chi^{2}$ distribution. A large $\chi^{2}$ value (with small *p* value $< 0.05$) indicates poor fit regression model. The last three (bottom) rows show the percentage of bubbles that are correctly identified at predicted probability $> 0.5 \left( {50\% } \right)$. Robust standard errors are given in parentheses. *p* values are given in brackets.*, **, and *** indicate significance at 10%, 5%, and 1% levels, respectively.

The lagged inflation has a positive coefficient, indicating that an increase in the variable is associated with a higher likelihood of bubble occurrence for precious metals. This is because metals can be considered an effective hedge against inflation. An appreciation in the USD value reduces the likelihood of bubbles because the wealth-saving attribute of precious metals declines with an increase in currency demand. The policy rate (EFR) coefficient is negative, suggesting that metal market bubbles are likely to occur with expansionary monetary policies to stimulate the economy (i.e., implementing a low policy rate).^19^ Consistent with the interest rate findings, the results also show a significant positive relationship between the global real economic activity index and bubbles. Therefore, an expansionary monetary policy increases the likelihood of bubbles.

Panel B shows the average marginal effects of the predictor variables. As shown, the marginal effects of a decrease in economic news-based sentiment increase the chance of a bubble in gold and platinum markets, and the effect of sentiment on silver and palladium price bubbles is not significant.

Table 9 presents the results using the MCSI and SIBW (panels A and B). The results are consistent with our earlier conclusion that bearish sentiment increases the likelihood of gold and platinum bubbles, with coefficients at − 0.931 and − 0.5110 (− 0.859 and − 0.287) in panels A and B respectively. The absolute value of the sentiment coefficient on gold is greater than that on platinum, thus indicating a more substantial economic significance of sentiment on the gold market.

**Table 9:** Probit results using alternative measures of sentiment

	Dependent variable: Bubble			
	Gold	Silver	Palladium	Platinum
*Panel A: Consumer Sentiment Index-(MCSI)*				
$MCSI_{t - 1}$	− 0.6237**	− 0.4250	0.0505	− 0.5351***
	(0.0320)	(0.2530)	(0.4390)	(0.0000)
$Inflation_{t - 1}$	0.0454**	0.0635*	0.0219***	0.0323***
	(0.0495)	(0.0768)	(0.0000)	(0.0000)
$US{\text{DI}}_{t - 1}$	− 0.6837***	− 0.7483**	− 0.6510*	0.1133
	(0.0000)	(0.0100)	(0.0970)	(0.2090)
${\text{EFR}}_{t - 1}$	− 0.1346*	0.2611	− 0.3583***	− 0.2892***
	(0.0820)	(0.2310)	(0.0000)	(0.0000)
$T - Spread_{t - 1}$	− 0.5169**	− 0.3037**	− 0.2870	− 0.4358**
	(0.0310)	(0.0100)	(0.5050)	(0.0170)
${\text{GEA}}_{t - 1}$	0.0055***	0.0060***	0.0014*	0.0578***
	(0.0010)	(0.0300)	(0.0680)	(0.0010)
$Constant$	0.5464***	0.8085***	0.9020***	0.5881***
	(0.0021)	(0.0000)	(0.0000)	(0.0000)
$Observations$	420	420	420	420
McFadden's pseud-R^2^	0.6464	0.5775	0.4357	0.5603
Log-likelihood	− 105.8980	− 47.6920	− 88.2729	− 159.7165
Hosmer–Lemeshow test	14.93	10.05	13.65	9.02
	(0.1019)	(0.2093)	(0.1006)	(0.2290)
*Panel B: Sentiment index in *Baker and Wurgler (2006)*-(SIBW)*				
$S{\text{SIBW}}_{t - 1}$	− 0.7794***	0.1973	− 0.0690	− 0.3565**
	(0.0060)	(0.7650)	(0.5930)	(0.0320)
$Inflation_{t - 1}$	0.2538***	0.0182***	0.0625***	0.0091***
	(0.0068)	(0.0093)	(0.0000)	(0.0000)
$US{\text{DI}}_{t - 1}$	− 1.0930***	− 0.9085***	− 1.0120***	0.0358
	(0.0000)	(0.0020)	(0.0094)	(0.1310)
${\text{EFR}}_{t - 1}$	− 0.1996**	− 0.1831**	− 0.2339***	− 0.8365***
	(0.0145)	(0.0035)	(0.0000)	(0.0000)
$T - Spread_{t - 1}$	− 0.6899***	− 1.1837**	− 0.3009*	− 0.4819*
	(0.0020)	(0.0180)	(0.0930)	(0.0690)
${\text{GEA}}_{t - 1}$	0.0046***	0.0062**	0.0057***	0.0072**
	(0.0010)	(0.0220)	(0.0000)	(0.0220)
$Constant$	0.4209***	0.5323***	0.4170***	0.8651***
	(0.0060)	(0.0002)	(0.0000)	(0.0000)
$Observations$	408	408	408	408
McFadden's pseud-R^2^	0.6803	0.5721	0.3365	0.4256
Log-likelihood	− 104.1921	− 48.2371	− 161.8798	− 84.6209
Hosmer–Lemeshow test	12.76	4.71	5.19	5.13
	(0.2371)	(0.8946)	(0.6050)	(0.6106)

This table reports the results using alternative measures of sentiment. Panels A and B report the results using the MCSI and SIBW, respectively. The dependent variable is a binary that equals 1 (bubble dates) and 0 (none-bubble dates) identified by the GSADF procedure. The Hosmer–Lemeshow test is a statistical test for goodness of fit for probit regressions, following the $\chi^{2}$ distribution. A large $\chi^{2}$ value (with small *p* value $< 0.05$) indicates poor fit regression model. Robust standard errors are given in parentheses. *p* values are given in brackets.*, **, and *** indicate significance at 10%, 5%, and 1% levels, respectively.

**Fig. 2:**
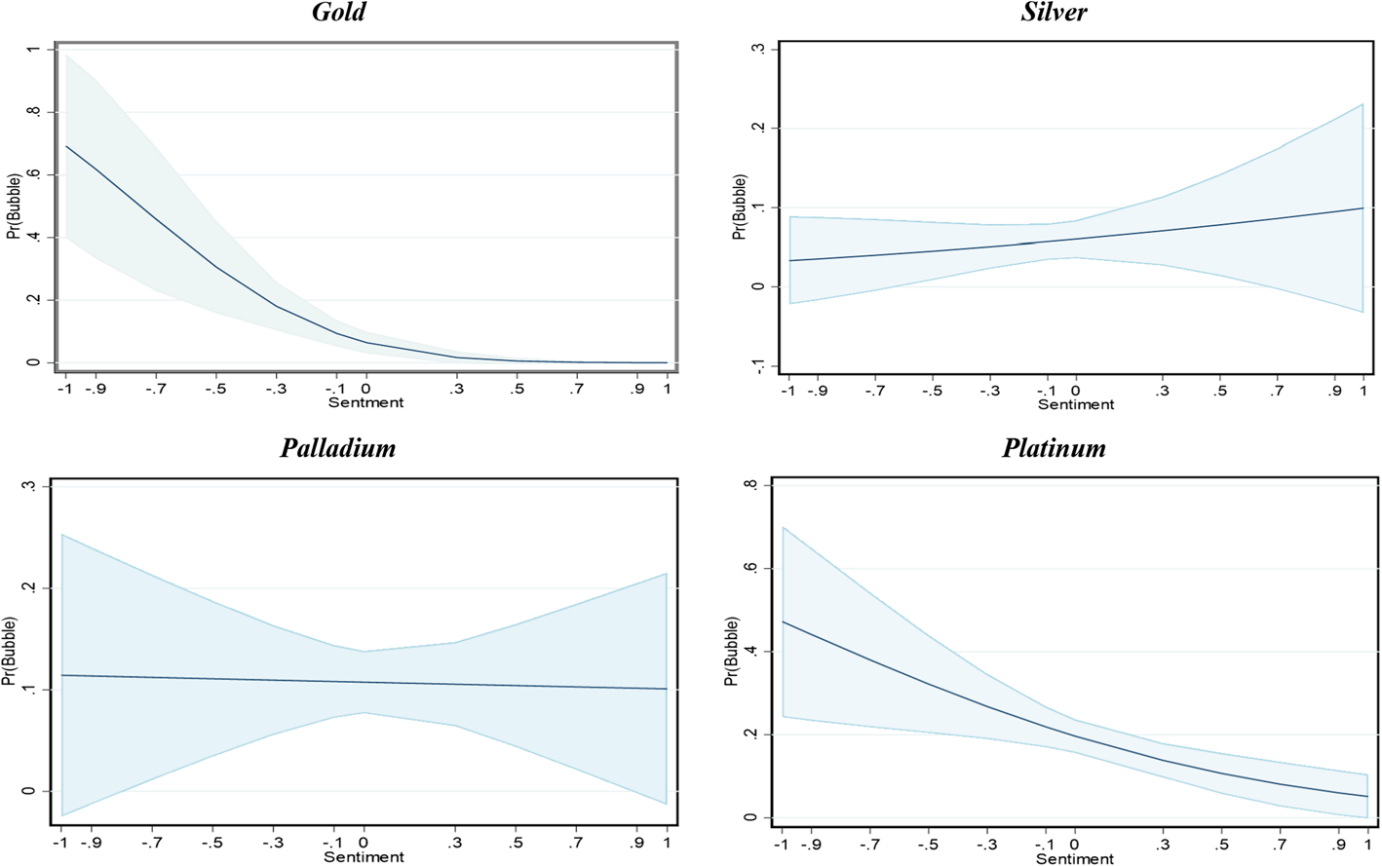
Marginal effects of sentiment. Note: The graphs show the marginal effects of the statistically significant news sentiment on the probability of a bubble (Table 7, Panel B) with 95% confidence intervals (blue areas). The y-axis shows a bubble's probability, and the x-axis show the news-based sentiment index

Figure 2 illustrates the marginal effects of sentiment on creating price bubbles for these metal commodities. The bearish sentiment (i.e., negative values) increases the probability of bubble occurrence for gold and platinum, thus supporting our findings in Tables 8 and 9. The largest effect of sentiment is observed when its value shifts from negative to positive. Conversely, positive sentiment slightly increases the probability of bubble occurrence for silver, although this effect is small. Finally, sentiment does not affect palladium bubbles since this metal is influenced by its supply and demand fundamentals, and financial factors, such as the USD value.

**Fig. 3:**
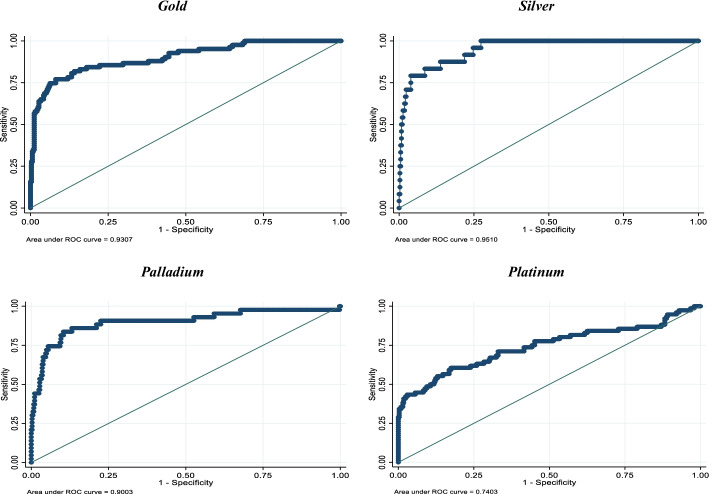
Receiver operating characteristic (ROC) curves. Notes: The graphs show the receiver operating characteristic (ROC) curves. A model with no predictive power has an AUC = 0.5; a perfect model has an AUC = 1

Figure 3 shows the prediction accuracy of sentiment on commodity price bubbles using the ROC curve, which shows that a model incorporating sentiment to predict gold and silver price bubbles outperforms—obtaining an under the curve (AUC) value of approximately 0.95—the prediction accuracy of the same model used to predict palladium (AUC = 0.90) and platinum (AUC = 0.74).

Overall, the findings are robust across different sentiment measures, indicating that sentiment is a successful predictor of gold and platinum. In particular, bearish sentiment increases the likelihood of bubble occurrence, and a model that incorporates sentiment into predicting precious metal bubbles can perform well.

### Robustness analysis

In this section, we check the robustness of our main findings to (a) an alternative indicator of sentiment, (b) structural break, and two subperiods (c) additional control variables.

### Market sentiment measure

The novelty of our study is the utilization of news-based economic sentiment. To check for robustness and to provide a complete comparison with the results found in the previous literature, we repeat our analysis using indirect market sentiment measures (i.e., the option implied volatility index [VIX]). The Chicago Board of Options Exchange (CBOE) constructed and published the VIX index. The VIX index is computed from the transacted option prices (i.e., out-of-the-money calls and puts)^20^; thus, it is a forward-looking measure of investor sentiment. This index has been used by many researchers as proxies for investor fear gauges and for tracking investor sentiment.^21^ The VIX index was obtained from January 1990 to August 2020 (totaling 360 observations). The data is derived from Thomson Reuters' Datastream according to its availability. Table 12 of the “Appendix” presents the descriptive statistics of the data.

Table 10 reports the results using the VIX index. The coefficients of the lagged VIX are positive and statistically significant for gold and platinum. These results indicate that when the VIX is rising (i.e., greater investor fears), the probability of creating bubbles for both gold and platinum increases, consistent with the findings of Pan (2018) for gold. These results also confirm our earlier findings based on news-based sentiments measures that the probability of bubbles in gold and platinum significantly increases when the bearish sentiment is increased.

**Table 10:** Probit results using option implied volatility index -(VIX)

	Dependent variable: Bubble			
	Gold	Silver	Palladium	Platinum
$VIX_{t - 1}$	0.0879***	− 0.0022	0.0315	0.0481***
	(0.0000)	(0.7500)	(0.1270)	(0.0040)
$Inflation_{t - 1}$	2.0117**	0.6214*	1.9999***	1.7865***
	(0.0340)	(0.0733)	(0.0010)	(0.0000)
$US{\text{DI}}_{t - 1}$	− 1.3535***	− 1.0177***	− 3.1302***	− 2.8887***
	(0.0000)	(0.0000)	(0.0010)	(0.0000)
${\text{EFR}}_{t - 1}$	− 0.1018**	− 0.2849**	− 0.2213***	− 0.5029***
	(0.0199)	(0.0380)	(0.0000)	(0.0000)
$T - Spread_{t - 1}$	− 0.4770**	− 0.6280*	− 0.1583	− 0.3606**
	(0.0137)	(0.0600)	(0.0520) ***	(0.0375)
${\text{GEA}}_{t - 1}$	0.0042***	0.0101**	0.0081**	0.0097***
	(0.0010)	(0.0281)	(0.0150)	(0.0010)
$Constant$	0.3928***	0.2285***	0.3282***	− 0.1095***
	(0.0000)	(0.0020)	(0.0067)	(0.0000)
$Observations$	360	360	360	360
McFadden's pseud-R^2^	0.6170	0.5523	0.3227	0.5950
Log-likelihood	− 95.451	− 79.9894	− 172.0924	− 83.1346
Hosmer–Lemeshow test	3.060	3.780	7.920*	5.770
	(0.3831)	(0.4361)	(0.0832)	(0.1450)

This table reports the results using the option implied volatility index-(VIX). The dependent variable is a binary that equals 1 (bubble dates) and 0 (none-bubble dates) identified by the GSADF procedure. The Hosmer–Lemeshow test is a statistical test for goodness of fit for probit regressions, following the $\chi^{2}$ distribution. A large $\chi^{2}$ value (with small *p* value $< 0.05$) indicates poor fit regression model. Robust standard errors are given in parentheses. *p* values are given in brackets.*, **, and *** indicate significance at 10%, 5%, and 1% levels, respectively.

### Structural break: two subperiods

We performed a subperiod analysis to determine whether the earlier results were robust over different periods. Following the stochastic breakpoint test provided in Table 5, we divided the sample into two subperiods (January 1985 to November 2007 (pre-GFC) and December 2007 to August 2020 (post-GFC)). The results of the probit regressions for the two subsamples are presented in Table 11. Comparisons based on the Pseudo-R^2^ values indicate that the model's explanatory power in the post-GFC period is stronger than it is in the pre-GFC period. For gold and platinum, the coefficients of news sentiment are negative and significant in the two subperiods.

**Table 11:** Estimation results under different subsamples

	Dependent variable: Bubble			
	Gold	Silver	Palladium	Platinum
*Panel A: Before GFC* (*January 1985 to November 2007)*				
$NESI_{t - 1}$	− 0.8726**	− 0.2250	0.4289	− 0.5202***
	(0.0198)	(0.2810)	(0.5010)	(0.0000)
$Inflation_{t - 1}$	1.0110***	1.3990*	0.6475***	2.7806**
	(0.0020)	(0.0600)	(0.0000)	(0.0150)
$US{\text{DI}}_{t - 1}$	− 0.7840	− 2.4983**	− 0.7669***	− 0.6896***
	(0.1660)	(0.0120)	(0.0040)	(0.0010)
${\text{EFR}}_{t - 1}$	− 0.0204	− 0.2623	− 0.1273	− 0.2918***
	(0.8410)	(0.1580)	(0.2210)	(0.0020)
$T - Spread_{t - 1}$	− 0.2347	− 1.6647**	− 2.7865***	− 1.2880**
	(0.6610)	(0.0253)	(0.0010)	(0.0260)
${\text{GEA}}_{t - 1}$	0.0010	0.0181**	0.0055	0.0099**
	(0.8460)	(0.0340)	(0.1930)	(0.0150)
$Constant$	1.0716	3.9195**	5.2929	4.0492
	(0.4670)	(0.0437)	(0.3401)	(0.1460)
$Observations$	272	272	272	272
McFadden's pseud-R^2^	0.3998	0.5996	0.3219	0.4873
Log-likelihood	− 54.1213	− 39.6921	− 68.1614	− 74.8537
Hosmer–Lemeshow test	3.17	3.97	12.29	9.88
	(0.9235)	(0.8595)	(0.1388)	90.2737)
Panel B: After GFC (December 2007 to August 2020)				
$NESI_{t - 1}$	− 2.7319***	− 1.2156***	0.5930	− 1.0272***
	(0.0019)	(0.0027)	(0.7240)	(0.0000)
$Inflation_{t - 1}$	2.1302***	2.4118*	1.7276**	3.1534***
	(0.0060)	(0.0703)	(0.0110)	(0.0000)
$US{\text{DI}}_{t - 1}$	− 5.4248*	− 4.8325***	− 1.3515	− 2.0290***
	(0.0590)	(0.0013)	(0.7320)	(0.0001)
${\text{EFR}}_{t - 1}$	− 1.2303**	− 1.2263**	− 2.1625*	− 0.9474***
	(0.0296)	(0.0380)	(0.0646)	(0.0000)
$T - Spread_{t - 1}$	− 1.2499***	− 4.2208***	− 0.6202	− 2.3639
	(0.0072)	(0.0029)	(0.5510)	(0.0038)
${\text{GEA}}_{t - 1}$	0.0203**	0.0146*	0.0042	0.0394***
	(0.0448)	(0.0512)	(0.5160)	(0.0094)
$Constant$	1.6553*	3.0170***	9.8635**	5.1021*
	(0.0660)	(0.0015)	(0.0140)	(0.0790)
$Observations$	136	136	136	136
McFadden's pseud-R^2^	0.9532	0.8488	0.3632	0.9607
Log-likelihood	− 14.1578	− 16.13851	− 25.8458	− 45.083
Hosmer–Lemeshow test	7.75	0.04	1.76	6.89
	(1.0000)	(1.0000)	(0.9874)	(0.4185)

The dependent variable is a binary that equals 1 (bubble dates) and 0 (none-bubble dates) identified by the GSADF procedure. Panels A and B report the results of the probit regressions based on the news-based economic sentiment index (NESI). The Hosmer–Lemeshow test is a statistical test for goodness of fit for probit regressions, which follows an $\chi^{2}$ distribution. A large $\chi^{2}$ value (with small *p* value $< 0.05$) indicates poor fit regression model. *p* values are given in brackets.*, **, and *** indicate significance at 10%, 5%, and 1% levels, respectively.

In contrast, the effect of sentiment on palladium price bubbles is not significant in the two subperiods. For silver, the effect of news sentiment on price bubbles is statistically significant only in the post-GFC period. Additionally, it is shown that the negative influence of news sentiment on gold and platinum bubbles is mostly observed in the post-GFC period. The same effect can be observed for silver with a sentiment coefficient of -1.21 in the post-GFC period and -0.22 in the pre-GFC period. Overall, the evidence suggests that the predictability of news sentiment on precious metal bubbles is more pronounced during bad economic conditions (i.e., crises). These findings indicate that low sentiment shifts a greater demand for safe-haven commodities (i.e., gold, silver, and platinum) during crises, thereby raising their prices and creating bubbles.

### Additional control variables

The robustness of the results is also checked by extending the model with additional control variables that measure uncertainty and infectious disease pandemics such as COVID-19. Therefore, we analyzed the impact of indicators of uncertainty and infectious disease epidemics on the probability of bubbles in precious metal markets. Several studies have shown that uncertainty and the eruption of COVID-19 have caused more significant fluctuations in commodity markets. For example, Balcilar et al. (2016), Beckmann et al. (2019), and Zhang et al. (2021) showed that various uncertainty measures (i.e., economic policy uncertainty) have significant explanatory power in commodity returns and volatility. Gozgor et al. (2019), Das et al. (2019), and Baur and Smales (2020) observed how GPR could play a role in precious metal returns. In addition, several recent studies confirm that the eruption of COVID-19 has considerably affected precious metal commodities (Yousaf 2021; Umar et al. 2021a, b; Salisu et al. 2021; Maghyereh and Abdoh 2022; among many others).

Following Beckmann et al. (2019), Gozgor et al. (2019), Zhang et al. (2021), and other recent studies, we use two different news-based indicators for uncertainty: the economic policy uncertainty (EPU) index of Baker et al. (2016)^22^ and the GPR index of Caldara and Iacoviello (2021). The EPU index is constructed based on newspaper coverage of three types of news: policy-related economic uncertainty, the number of federal tax code provisions set to expire, and the disagreement among professional forecasters on future tendencies of relevant macroeconomic variables. The GPR index was constructed based on newspaper articles covering geopolitical tensions, wars, and terrorist acts.^23^

To quantitative impacts of the infectious disease pandemic (i.e., such as the COVID-19) on the probability of the occurrence of bubbles in precious metal markets, we use the Infectious Disease Equity Market Volatility Tracker (ID-EMV) recently developed by Baker et al. (2020a,b). This is a newspaper-based index developed using machine learning-based textual analysis with a higher level of the index, indicating a higher level of pandemic uncertainty in the financial markets.^24^ Data on the EPU, GPR, and ID-EMV indices covering January 1985 to August 2020 was downloaded from https://www.policyuncertainty.com/.^25^ Table 12 of the “Appendix” presents the descriptive statistics of the data.

Table 13 of the “Appendix” reports the results of the logistic model with the additional control variables. The results show that Economic Policy Uncertainty (EPU) is positive and significant for Gold, Palladium, and Platinum, indicating that the probability of creating bubbles for these metals increases during uncertain times. This finding coincides with the results of Balcilar et al. (2016), Beckmann et al. (2019), and Zhang et al. (2021) who found evidence that economic policy uncertainty has significant explanatory power in commodity returns and volatility.

Similarly, the results show that the GPR increases the chances of observing bubbles for gold, palladium, and platinum. These findings suggest that during geopolitical threats, higher GPR shifts a greater demand for safe-haven commodities (i.e., gold, palladium, and platinum), raising their prices and creating bubbles. This is consistent with the findings of Baur and Smales (2020), who argue that precious metals can hedge against GPR.

Finally, the Infectious Disease Equity Market Volatility tracker (ID-EMV) increases the likelihood of a gold price bubble but does not significantly affect the bubbles of other metals. This finding controverts our earlier conclusion of the greater safe-haven role of gold compared to other precious metals during the infectious disease pandemic (i.e., COVID-19 pandemic).

One observation of the results is that, the coefficients of lagged NESI are significant predictors of gold and platinum, but with a different coefficient sign. This finding suggests that EPU, GPR, and ID-EMV indices directly affect sentiment, subsuming the negative effect of NESI on metal price bubble occurrence.^26^

## Conclusions

We examine whether sentiment can capture the price bubbles of four important precious metals, namely; gold, silver, platinum, and palladium, as explained by the standard inflation indicators, the Fed's policy rate, and the USD value. We employed the SADF and GSADF approaches to discern the price bubbles. Our findings imply that gold and platinum's bubbles predictability improve when market sentiment is considered, and bearish sentiments increase the likelihood of bubble occurrence. We test the robustness of these findings using three measures of sentiment; Shapiro, Hale, Sudhof, and Wilson’s (2020) NESI, the MCSI, and SIBW. Furthermore, we date-stamp the bubble episodes and find that they correspond to the 2008–2009 global financial crisis and the 2010 European sovereign debt crisis. Nevertheless, gold is the only metal that has witnessed bubble episodes during the COVID-19 pandemic.

Our findings have implications for both policymakers and investors. Global producers and consumers of precious metals can utilize these findings to detect bubbles and immediately mitigate price risks. Investors can avoid making long positions when the metal is in a bubble episode, whereas they can make short positions before the bubble bursts or its price declines substantially.

## Data Availability

All data are obtained via individual data channels such as the Thomson Reuters Datastream database, Federal Reserve Bank of St. Louis, and Federal Reserve Bank of San Francisco. The models and data analysis are applied through computer software such as MATLAB, R, and Stata. All data and codes will be available from the authors upon request upon request.

## Appendix

See Tables

**Table 12:** Descriptive statistics of additional variables

	VIX	EPU	GPR	ID-EMV
*Panel A: Summary descriptive statistics*				
Mean	19.2933	111.7145	86.0089	28.2249
Median	17.4350	104.1809	66.3031	11.5800
Maximum	62.9800	350.4598	545.2632	1556.6800
Minimum	9.4500	57.2026	23.7440	0.0000
Std. Dev	7.6740	37.6639	63.7495	116.3420
Skewness	1.8874	1.8532	2.9797	9.5788
Kurtosis	8.4230	9.3209	16.3471	103.6355
Jarque–Bera test	669.4***	957.5***	3810.2***	187,152.3***
	(0.0000)	(0.0000)	(0.0000)	(0.0000)

	ADF test		PP test	
	Including intercept	Including intercept and trend	Including intercept	Including intercept and trend
*Panel B: ADF and PP stationary tests*				
VIX	− 6.6571***	− 6.6965***	− 6.3345***	− 6.3841***
	(0.0000)	(0.0000)	(0.0000)	(0.0000)
EPU	− 2.7697*	− 3.1889*	− 5.9159***	− 6.4246***
	(0.0635)	(0.0880)	(0.0000)	(0.0000)
GPR	− 7.7869***	− 8.3431***	− 7.9478***	− 8.5735***
	(0.0000)	(0.0000)	(0.0000)	(0.0000)
ID-EMV	− 5.4481***	− 5.7126***	− 5.3729***	− 5.6567***
	(0.0000)	(0.0000)	(0.0000)	(0.0000)

	Including intercept		Including intercept and trend	
	Test statistics	Break date	Test statistics	Break date
*Panel C**: **Zivot-Andrews (ZA) stationary test*				
VIX	− 4.6920**	2011M11	− 4.8333**	2008M10
	(0.0106)		(0.0440)	
EPU	− 4.0869**	2007M08	− 4.2335*	2007M08
	(0.0408)		(0.0755)	
GPR	− 5.0922*	2001M09	− 5.1344*	2001M09
	(0.0731)		(0.0908)	
ID-EMV	− 5.9350	2015M04	− 7.5298**	2015M04
	(0.1550)		(0.0199)	

The Zivot-Andrews (ZA) which allows for both a structural break in intercept, trend or both. The null hypothesis of the ZA test is that the series has a unit root with a structural break(s) against the alternative hypothesis that they are stationary with a break(s). *p* values are given in brackets. *, **, and *** indicate significance at 10%, 5%, and 1% levels, respectively.

12 and

**Table 13:** Probit results with additional control variables

	Dependent variable: Bubble			
	Gold	Silver	Palladium	Platinum
$NESI_{t - 1}$	0.0702**	0.0036	0.0206	0.0474***
	(0.0154)	(0.7690)	(0.7400)	(0.0000)
$Inflation_{t - 1}$	1.6046*	0.6159*	1.3735***	1.8759***
	(0.0940)	(0.0977)	(0.0000)	(0.0080)
$US{\text{DI}}_{t - 1}$	− 1.6744**	− 1.6348***	− 2.8399***	− 1.8218***
	(0.0010)	(0.0000)	(0.0000)	(0.0049)
${\text{EFR}}_{t - 1}$	− 0.0031*	− 0.2431*	− 0.5383***	− 0.1161***
	(0.0970)	(0.0890)	(0.0000)	(0.0020)
$T - Spread_{t - 1}$	− 0.0479*	− 0.4905*	− 0.2297*	− 0.3733**
	(0.0887)	(0.0680)	(0.0519)	(0.0235)
${\text{GEA}}_{t - 1}$	0.0083***	0.0070**	0.0046***	0.0049***
	(0.0000)	(0.0260)	(0.0032)	(0.0030)
${\text{EPU}}_{t - 1}$	0.0128**	0.0037	0.0045**	0.0264***
	(0.0130)	(0.6130)	(0.0362)	(0.0000)
${\text{GPR}}_{t - 1}$	0.0086***	0.0014	0.0120***	0.0006**
	(0.0070)	(0.7910)	(0.0000)	(0.0409)
${\text{ID}} - {\text{EMV}}_{t - 1}$	0.0254***	− 0.0094	0.0012	0.0040
	(0.0000)	(0.7280)	(0.9150)	(0.6020)
$Constant$	0.6003	0.1959**	0.7914***	− 0.1788*
	(0.1560)	(0.0120)	(0.0000)	(0.0554)
$Observations$	408	408	408	408
McFadden's pseud-R^2^	0.7546	0.4845	0.4657	0.6786
Log-likelihood	− 88.0272	− 67.0566	− 144.0302	− 85.3862
Hosmer–Lemeshow test	6.030	1.570	2.250	5.960
	(0.4193)	(0.6670)	(0.1332)	(0.1508)

The dependent variable is a binary that equals 1 (bubble dates) and 0 (none-bubble dates) identified by the GSADF procedure. The Hosmer–Lemeshow test is a statistical test for goodness of fit for probit regressions, following the $\chi^{2}$ distribution. A large $\chi^{2}$ value (with small *p* value $< 0.05$) indicates poor fit regression model. *p* values are given in brackets.*, **, and *** indicate significance at 10%, 5%, and 1% levels, respectively.

13.

## Abbreviations

VIX: Volatility index
ROC: Receiver operating characteristic curve
SADF: Supremum augmented Dickey–Fuller
GSADF: Generalized SADF
BSADF: Backward SADF
NESI: News-based economic sentiment index
MCSI: Michigan Consumer Sentiment Index
SIBW: Investor sentiment index
ADF: Augmented Dickey–Fuller test
PP: Phillips–Perron test

## Footnotes

1 Other works of Wang et al. (2018), Wang et al. (2020), Umar et al. (2021a, b), Wang et al. (2021), Caferra et al. (2021), among others, examine bubbles in various markets such as food, oil, and bitcoin.

2 The volatility index is viewed as a "fear gauge" or "fear index" among market participants. It is constructed based on out-of-money put and calls S&P 500 index options prices using a near-term option contract and a next-term option contract. Therefore, it is determined by the sophisticated trades of the index option market (John and Li 2021).

3 Algaba et al. (2020) provide a survey of the methodology and applications related to an econometric analysis of sentiment extracted from text data.

4 We further used the option implied volatility index (VIX) for robustness checks.

5 See Gürkaynak (2008) for a comprehensive review of these methods.

6 Homm and Breitung (2012) conducted simulation studies to compare the methods used to test for speculative bubbles (i.e., Bhargava statistic, Busetti–Taylor statistic, Kim statistic, and PWY). They found that the PWY method is more robust than other methods.

7 The GSADF has recently become the most widely used method in literature for detecting real-time financial bubbles. See, for example, Phillips and Shi (2019a, 2020) for a comprehensive literature review.

8 The multiplicity problem refers to the probability of making a false positive conclusion (type 1 error, the rejection of a true null hypothesis), which rises with the number of hypotheses in a test (Shi et al. 2018; Phillips and Shi 2018, 2020).

9 Expected future capital gains reflect changes in current and expected future market conditions.

10 Under the null hypothesis of normal market conditions, the PSY statistic has the following limit distribution (PSY 2015a): $\underbrace {\sup }_{{r_{1} \in \left[ {0,r_{2} - r_{0} } \right],r_{2} = \left[ {r_{0} ,1} \right]}}\left\{ {\frac{{\frac{1}{2}r_{w} \left[ {W\left( {r_{2} } \right)^{2} - W\left( {r_{1} } \right)^{2} - r_{w} } \right] - \mathop \smallint \nolimits_{{r_{1} }}^{{r_{3} }} W\left( s \right)ds\left[ {W\left( {r_{2} } \right) - W\left( {r_{1} } \right)} \right]}}{{r_{w}^{1/2} \left[ {r_{w} \mathop \smallint \nolimits_{{r_{1} }}^{{r_{2} }} W\left( s \right)^{2} ds - \left( {\mathop \smallint \nolimits_{{r_{1} }}^{{r_{3} }} W\left( s \right)ds} \right)^{2} } \right]^{1/2} }}} \right\}$, where $r_{w} = r_{2} - r_{2}$ and $W\left( \cdot \right)$ are the standard Wiener process.

11 We used the “psymonitor” R package for implementing the GSADF test and bootstrap procedure. The “psymonitor” R package can be found at: https://github.com/itamarcaspi/psymonitor.

12 The flight-to-safety effect is referred to as the act of shifting from “risky” investments to “safe” investments during periods of economic uncertainty.

13 Additionally, Hammoudeh et al. (2015) and Huang et al. (2019) provided evidence that interest rate (measured by the Fed's policy rate) decreases raises the gold price.

14 The index is provided regularly on the San Francisco Fed’s website: https://www.frbsf.org/economicresearch/indicators-data/daily-news-sentiment-index/

15 Shapiro et al. (2020) assessed the accuracy of their model and found that it performed better than other models that were constructed using machine learning techniques.

16 Additional information about the index can be found at: http://www.sca.isr.umich.edu/

17 Baker and Wurgler (2006) apply six stock market market-based proxies for sentiment, including stock trading volume, mutual fund flows, a number of initial public offerings (IPOs), average first-day IPO returns, equity share in new issues, and dividend premium. .

18 Baur and McDermott (2010) argued that gold acts as a safe-haven asset for financial markets. Therefore, we believe gold has the greatest frequency of bubble episodes as investors resort to gold during crises (e.g., the global financial crises and European sovereign debt crises).

19 For example, see Zhao et al. (2015).

20 For the calculation of VIX index, see the CBOE's "VIX white paper" at https://www.cboe.com/micro/vix/vixwhite.pdf.

21 For example, see Dennis et al. (2006), Awartani et al. (2016), Maghyereh et al. (2016, 2021), Fassas and Siriopoulos (2021).

22 We also ran a robustness check using the uncertainty index (i.e., measures macroeconomic uncertainty) developed by Jurado et al. (2015). We found that our results (the results are not provided because of space limitations but are available upon request from the authors) do not significantly change.

23 Caldara and Iacoviello (2021) and Baur and Smales (2020) provide evidence that the GPR index captures a risk factor that is different to other policy, political, and financial market uncertainty measures.

24 For detailed information on the EMVT-ID index, see https://www.policyuncertainty.com/infectious_EMV.html.

25 Data on the ID-EMV is only available at a daily basis. Following Bai et al. (2021), we sum the daily EMV-ID records to get the monthly EMV-ID index within a month each month.

26 We thank an anonymous reviewer for pointing these observations out.

## References

[CR1] Adrangi B, Chatrath A, Raffiee K (2003). Economic activity, inflation, and hedging: The case of gold and silver investments. J Wealth Manag.

[CR2] Agnello L, Castro V, Hammoudeh S, Sousa RM (2020) Global factors, uncertainty, weather conditions and energy prices: on the drivers of the duration of commodity price cycle phases. Energy Econ 90:104862

[CR3] Aguilar P, Ghirelli C, Pacce M, Urtasun A (2021). Can news help measure economic sentiment? An application in COVID-19 times. Econ Lett.

[CR4] Algaba A, Ardia D, Bluteau K, Borms S, Boudt K (2020). Econometrics meets sentiment: an overview of methodology and applications. J Econ Surv.

[CR5] Ang A, Piazzesi M, Wei M (2006). What does the yield curve tell us about GDP growth?. J Econom.

[CR6] Arango LE, Arias F, Florez A (2012). Determinants of commodity prices. Appl Econ.

[CR7] Areal FJ, Balcombe K, Rapsomanikis G (2016). Testing for bubbles in agriculture commodity markets. Economia Agraria y Recursos Naturales.

[CR8] Awartani B, Maghyereh A, Cherif G (2016). The connectedness between crude oil and financial markets: evidence from implied volatility indices. J Commod Mark.

[CR9] Awartani B, Maghyereh A, Ayton J (2020). Oil price changes and industrial output in the MENA region: nonlinearities and asymmetries. Energy.

[CR10] Bai L, Wei Y, Wei G, Li X, Zhang S (2021). Infectious disease pandemic and permanent volatility of international stock markets: a long-term perspective. Finance Res Lett.

[CR11] Baker M, Wurgler J (2006). Investor sentiment and the cross-section of stock returns. J Financ.

[CR12] Baker M, Wurgler J (2007). Investor sentiment in the stock market. J Econ Perspect.

[CR13] Baker M, Wurgler J, Yuan Y (2012). Global, local, and contagious investor sentiment. J Financ Econ.

[CR14] Baker S, Bloom N, Davis S (2016). Measuring economic policy uncertainty. Quart J Econ.

[CR15] Baker SR, Bloom N, Davis SJ, Kost K, Sammon M, Viratyosin T (2020a) The unprecedented stock market reaction to COVID-19. White paper-Becker Friedman Institute for economics at UChicago, pp. 1–12. https://stockmarketjumps.com/files/COVIDMarketReaction.pdf.

[CR16] Baker S, Bloom N, Davis SJ, Terry SJ (2020b) COVID-induced economic uncertainty. National Bureau of Economic Research, No. w26983

[CR17] Balcilar M, Gupta R, Pierdzioch C (2016). Does uncertainty move the gold price? New evidence from a nonparametric causality-in-quantiles test. Resour Policy.

[CR18] Bampinas G, Panagiotidis T (2015). Are gold and silver a hedge against inflation? A two century perspective. Int Rev Financ Anal.

[CR19] Batten JA, Ciner C, Lucey BM (2010). The macroeconomic determinants of volatility in precious metals markets. Resour Policy.

[CR20] Baur DG, McDermott TK (2010). Is gold a safe haven? International evidence. J Bank Finance.

[CR21] Baur DG, Smales LA (2020). Hedging geopolitical risk with precious metals. J Bank Finance.

[CR22] Baur D, Tran D (2014). The long-run relationship of gold and silver and the influence of bubbles and financial crises. Empir Econ.

[CR23] Beckmann J, Berger T, Czudaj R (2019). Gold price dynamics and the role of uncertainty. Quant Finance.

[CR24] Bedoui R, Guesmi K, Kalai S, Porcher T (2020). Diamonds versus precious metals: What gleams most against USD exchange rates?. Finance Res Lett.

[CR25] Brochado A (2020). Google search-based sentiment indexes. IIMB Manag Rev.

[CR26] Brown GW, Cliff MT (2004). Investor sentiment and the near-term stock market. J Empir Financ.

[CR27] Buckman SR, Shapiro AH, Sudhof M, Wilson DJ (2020). News Sentiment in the Time of COVID-19. FRBSF Econ Lett.

[CR28] Caferra R, Tedeschi G, Morone A (2021). Bitcoin: Bubble that bursts or gold that glitters?. Econ Lett.

[CR29] Caldara D, Iacoviello M (2021) Measuring geopolitical risk. Working paper, Board of Governors of the Federal Reserve. https://www.matteoiacoviello.com/gpr_files/GPR_PAPER.pdf

[CR30] Capie F, Mills TC, Wood G (2005). Gold as a hedge against the dollar. J Int Finan Markets Inst Money.

[CR31] Collin-Dufresne P, Goldstein RS, Martin JS (2001). The determinants of credit spread changes. J Finance.

[CR32] Das D, Kannadhasan M, Bhowmik P (2019). Geopolitical risk and precious metals. J Econ Res.

[CR33] Dennis P, Mayhew S, Stivers C (2006). Stock returns, implied volatility innovations, and the asymmetric volatility phenomenon. J Financ Quant Anal.

[CR34] Diba BT, Grossman HI (1988). Explosive rational bubbles in stock prices. Am Econ Rev.

[CR35] Doblas-Madrid A (2012). A robust model of bubbles with multidimensional uncertainty. Econometrica.

[CR36] Duarte AM, Gaglianon WP, Guillén O, Issler JV (2021). Commodity prices and global economic activity: a derived-demand approach. Energy Econ.

[CR37] Fassas AP, Siriopoulos C (2021). Implied volatility indices—a review. Q Rev Econ Finance.

[CR38] Fernandez V (2016). Some facts on the platinum-group elements. Int Rev Financ Anal.

[CR39] Frankel JA, Campbell J (2006). The effect of monetary policy on real commodity prices. Asset prices and monetary policy.

[CR40] Frankel JA (2014). Effects of speculation and interest rates in a “carry trade” model of commodity prices. J Int Money Financ.

[CR41] Froot K, Obstfeld M (1991). Intrinsic bubbles: the case of stock prices. Am Econ Rev.

[CR42] Gao L, Süssb S (2015). Market sentiment in commodity futures returns. J Empir Financ.

[CR43] Gharib C, Mefteh-Wali S, Ben Jabeur S (2021). The bubble contagion effect of COVID-19 outbreak: Evidence from crude oil and gold markets. Finance Res Lett.

[CR500] Gozgor G, Lau CKM, Sheng M, Yarovaya L (2019) The role of uncertainty measures on the returns of gold. Econ Lett 185:108680

[CR44] Gruber, J.W. & Vigfusson, R.J. (2013). Do low interest rates decrease commodity price volatility? Retrieved from Board of Governors of the Federal Reserve System (US).

[CR45] Gurkaynak RS (2008). Econometric tests of asset price bubbles: taking stock. J Econ Surv.

[CR46] Hammoudeh S, Nguyen DK, Sousa RM (2015). US monetary policy and sectoral commodity prices. J Int Money Financ.

[CR47] Harmston S (1998) Gold as a store of value. Research Study No. 22. The World Gold Council. London, ZDB-ID 2202126–7, vol 22

[CR48] Harris RDF, Shen J (2017). The intrinsic value of gold: An exchange rate-free price index. J Int Money Finance.

[CR49] Harvey DI, Leybourne SJ, Sollis R, Taylor AMR (2016). Tests for explosive financial bubbles in the presence of non-stationary volatility. J Empir Financ.

[CR50] Harvey DI, Leybourne SJ, Zu Y (2019). Sign-based unit root tests for explosive financial bubbles in the presence of nonstationary volatility. Economet Theor.

[CR51] Harvey DI, Leybourne SJ, Zu Y (2020). Sign-based unit root tests for explosive financial bubbles in the presence of deterministically time-varying volatility. Economet Theor.

[CR52] Hau H, Rey H (2006). Exchange rates, equity prices and capital flows. Rev Financ Stud.

[CR53] Hillier D, Draper P, Faff R (2006). Do precious metals shine? An investment perspective. Financ Anal J.

[CR54] Homm U, Breitung J (2012). Testing for speculative bubbles in stock markets: a comparison of alternative methods. J Financ Economet.

[CR55] Huang X, Jia F, Xu X (2019). The threshold effect of market sentiment and inflation expectations on gold price. Resour Policy.

[CR56] John K, Li J (2021). COVID-19, volatility dynamics, and sentiment trading. J Bank Finance.

[CR57] Jurado K, Ludvigson SC, Ng S (2015). Measuring uncertainty. Am Econ Rev.

[CR58] Karali B, Power GJ (2013). Short- and long-run determinants of commodity price volatility. Am J Agric Econ.

[CR59] Khalifa AA, Miao H, Ramchander S (2011). Return distributions and volatility forecasting in metal futures markets: Evidence from gold, silver, and copper. J Futur Mark.

[CR60] Kilian L (2009). Not all oil price shocks are alike: Disentangling demand and supply shocks in the crude oil market. Am Econ Rev.

[CR61] Kilian L (2019). Measuring global real economic activity: do recent critiques hold up to scrutiny?. Econ Lett.

[CR62] Kumar A, Lee CMC (2006). Retail investor sentiment and return comovements. J Financ.

[CR63] Kurov A (2010). Investor sentiment and the stock market’s reaction to monetary policy. J Bank Finance.

[CR64] Labate J (1994) How to hedge against inflation. Fortune, December, pp 56–57.

[CR65] Liu P, Tang K (2010) Bubbles in the commodity asset class: Detection and sources. Working paper. Center for Real Estate and Finance, Cornell University

[CR66] Lucas RE (1978). Asset prices in an exchange economy. Econometrica.

[CR67] Lucey BM, Li S (2015). What precious metals act as safe havens, and when? Some US Evidence. Appl Econ Lett.

[CR68] Lucey BM, O’Connor FA (2013). Do bubbles occur in the gold price? An investigation of gold lease rates and Markov Switching models. Borsa Istanbul Rev.

[CR69] Maghyereh A, Abdoh H (2020). The tail dependence structure between investor sentiment and commodity markets. Resour Policy.

[CR72] Maghyereh A, Abdoh H (2022) COVID-19 pandemic and volatility interdependence between gold and financial assets. Appl Econ 54(13):1473–1486

[CR70] Maghyereh AI, Awartani B, Bouri E (2016). The directional volatility connectedness between crude oil and equity markets: new evidence from implied volatility indexes. Energy Econ.

[CR71] Maghyereh A, Abdoh H, Al-Shboul M (2020). The impact of sentiment on commodity return and volatility. Rev Pac Basin Financ Mark Policies.

[CR73] Maghyereh A, Abdoh H, Awartani B (2021) Have returns and volatilities for financial assets responded to implied volatility during the COVID-19 pandemic? J Commodity Mark. 10.1016/j.jcomm.2021.100194

[CR74] Magrini E, Donmez A (2013) Agricultural commodity price volatility and its macroeconomic determinants: a GARCH-MIDAS approach. Publications Office of the European Union; 2013. JRC84138

[CR201] Mensi W, Hammoudeh S, Rehman MU, Al-Maadid AAS, Kang SH (2020) Dynamic risk spillovers and portfolio risk management between precious metals and global foreign exchange markets. N Am J Econ Finance 51:101086

[CR75] Mo D, Gupta R, Li B, Singh T (2018). The macroeconomic determinants of commodity futures volatility: evidence from Chinese and Indian markets. Econ Model.

[CR76] Monschang V, Wilfling B (2021) Sup-ADF-style bubble-detection methods under test. Empir Econ 61:145–172

[CR77] Nazlioglu S (2011). World oil and agricultural commodity prices: evidence from nonlinear causality. Energy Policy.

[CR78] Pan WF (2018). Sentiment and asset price bubble in the precious metals markets. Financ Res Lett.

[CR79] Pavlidis EG, Paya I, Peel DA (2017). Testing for speculative bubbles using spot and forward prices. Int Econ Rev.

[CR80] Pavlidis EG, Paya I, Peel DA (2018). Using market expectations to test for speculative bubbles in the crude oil market. J Money Credit Bank.

[CR81] Phillips PC, Shi SP (2018). Financial bubble implosion and reverse regression. Economet Theor.

[CR82] Phillips PCB, Shi S (2019). Detecting financial collapse and ballooning sovereign risk. Oxford Bull Econ Stat.

[CR83] Phillips PCB, Shi S (2019b) Online Supplement to the Paper: Detecting Financial Collapse and Ballooning Sovereign Risk, Technical report

[CR84] Phillips PCB, Shi S (2020). Real time monitoring of asset markets: bubbles and crises. Handbook Statist.

[CR85] Phillips PC, Wu Y, Yu J (2011). Explosive behavior in the 1990s Nasdaq: when did exuberance escalate asset values?. Int Econ Rev.

[CR86] Phillips PCB, Shi S, Yu J (2015). Testing for multiple bubbles: Historical episodes of exuberance and collapse in the S&P 500. Int Econ Rev.

[CR87] Phillips PCB, Shi S, Yu J (2015). Testing for multiple bubbles: Limit theory of real-time detectors’. Int Econ Rev.

[CR88] Pierdzioch C, Risse M, Rohloff S (2016). Are precious metals a hedge against exchange-rate movements? An empirical exploration using Bayesian additive regression trees. N Am J Econ Finance.

[CR89] Pierre P (1989). The great crash, the oil price shock, and the unit root hypothesis. Econometrica.

[CR90] Pineiro-Chousa J, Lopez-Cabarcos MA, Perez-Pico AM, Ribeiro-Navarrete B (2018). Does social network sentiment influence the relationship between the S&P500 and gold returns?. Int Rev Financ Anal.

[CR91] Pukthuanthong K, Roll R (2011). Gold and the dollar (and the Euro, Pound, and Yen). J Bank Finance.

[CR92] Regenwetter M, Davis-Stober CP (2018). The role of independence and stationarity in probabilistic models of binary choice. J Behav Decis Mak.

[CR93] Salisu AA, Ndako UB, Oloko TF (2019). Assessing the inflation hedging of gold and palladium in OECD countries. Resour Policy.

[CR94] Salisu AA, Raheem ID, Vo XV (2021). Assessing the safe haven property of the gold market during COVID-19 pandemic. Int Rev Financ Anal.

[CR95] Shahzad SJH, Raza N, Balcilar M, Ali S, Shahbaz M (2017). Can economic policy uncertainty and investors sentiment predict commodities returns and volatility?. Resour Policy.

[CR96] Shapiro AH, Sudhof M, Wilson DJ (2020) Measuring news sentiment. J Econom (in press)

[CR97] Shen J, Najand M, Dong F, He W (2017). News and social media emotions in the commodity market. Rev Behav Finance.

[CR98] Shi S, Phillips PCB, Hurn S (2018). Change detection and the causal impact of the yield curve. J Time Ser Anal.

[CR99] Shiller RJ, Fischer S, Friedman BM (1984). Stock prices and social dynamics. Brook Pap Econ Act.

[CR100] Sjaastad LA, Scacciavillani F (1996). The price of gold and the exchange rate. J Int Money Financ.

[CR101] Smales LA (2014). News sentiment in the gold futures market. J Bank Finance.

[CR102] Smales LA (2015). Asymmetric volatility response to news sentiment in gold futures. J Int Finan Markets Inst Money.

[CR103] Stiglitz JE (1990). Symposium on bubbles. J Econ Perspect.

[CR104] Taylor NJ (1998). Precious metals and inflation. Appl Financ Econ.

[CR105] Thorbecke W, Zhang H (2009). Monetary policy surprises and interest rates: Choosing between the inflation-revelation and excess sensitivity hypotheses. South Econ J.

[CR106] Tirole J (1985). Asset bubbles and overlapping generations. Econometrica.

[CR107] Tully E, Lucey BM (2007). A power GARCH examination of the gold market research. Res Int Bus Financ.

[CR108] Umar M, Su C-W, Rizvi SKA, Lobont O-R (2021). Driven by fundamentals or exploded by emotions: Detecting bubbles in oil prices. Energy.

[CR109] Umar Z, Aziz S, Tawil D (2021). The impact of COVID-19 induced panic on the return and volatility of precious metals. J Behav Exp Finance.

[CR110] Varadi VK (2013) Determinants of volatile commodity prices. Retrieved from SSRN Electronic Journal. Available at SSRN: https://ssrn.com/abstract=2201080 or 10.2139/ssrn.2201080

[CR112] Wang X-Q, Su C-W, Tao R, Lobont O-R (2018) When will food price bubbles burst? A review. Agric Econ 64(12):566–573

[CR111] Wang KH, Su CW, Tao R, Hao LN (2020). Are there periodically collapsing bubble behaviours in the global coffee market?. Agric Econ Res Policy Pract Southern Africa.

[CR113] Yang C, Li J (2013). Investor sentiment, information and asset pricing model. Econ Model.

[CR114] Yousaf I (2021). Risk transmission from the COVID-19 to metals and energy markets. Resour Policy.

[CR115] Zhang H, Demirer R, Huang J, Huang W, Suleman MT (2021). Economic policy uncertainty and gold return dynamics: evidence from high-frequency data. Resour Policy.

[CR116] Zhao Y, Chang HL, Su CW, Nian R (2015). Gold bubbles: when are they most likely to occur?. Jpn World Econ.

[CR117] Zheng Y (2015). The linkage between aggregate investor sentiment and metal futures returns: a nonlinear approach. Q Rev Econ Finance.

[CR118] Zhu YH, Fan JW, Tucker J (2018). The impact of monetary policy on gold price dynamics. Res Int Bus Financ.

[CR119] Zivot E, Andrews WK (1992). Further evidence on the great crash, the oil-price shock, and the unit root hypothesis. J Bus Econ Stat.

